# When Losses Matter More: A Dynamic Reference‐Dependent Evaluation of Suicide Risk

**DOI:** 10.1111/risa.70334

**Published:** 2026-09-29

**Authors:** Mamata Parhi, Jishu Dey, Ramzi Benkraiem, Ranadeva Jayasekera, Tapas Mishra

**Affiliations:** ^1^ Roehampton University London UK; ^2^ University of Southampton UK; ^3^ Audencia Business School Nantes France; ^4^ Trinity College, University of Dublin Ireland; ^5^ Vilnius University Vilnius Lithuania

**Keywords:** cumulative prospect theory, decision under uncertainty, loss aversion, suicidal ideation, South Korea

## Abstract

How do individuals transition from distress to suicidal ideation? Existing economic and epidemiological approaches typically model suicide risk as a function of expected outcomes or accumulated stressors, but they offer limited insight into how individuals evaluate worsening life circumstances under uncertainty. In this paper, we develop a dynamic, reference‐dependent model of suicidal ideation grounded in cumulative prospect theory. The model captures two behavioral mechanisms: (i) outcomes are evaluated relative to a shifting personal reference point, and (ii) perceived probabilities are transformed into nonlinear decision weights when individuals evaluate uncertain future states. Using longitudinal household‐level data from South Korea and a hierarchical Bayesian estimation strategy, we estimate individual‐level parameters governing loss aversion, probability weighting, and reference‐point adaptation. We find that transitions into suicidal ideation are significantly associated with entry into the loss domain relative to one's own prior well‐being, rather than with absolute income or health alone. Loss‐side sensitivity, rather than gain‐side valuation, shows the strongest association with ideation, especially following recent negative well‐being changes. These findings suggest that suicidal ideation is closely linked to how individuals perceive and process deteriorating conditions, rather than to those conditions alone. By integrating behavioral decision theory with longitudinal data, this study provides evidence consistent with a reference‐dependent loss‐processing pathway under uncertainty and helps explain vulnerability to extreme psychological outcomes.

## 1 Introduction

*“Losses often loom larger than gains”* (Kahneman and Tversky 1979)

The causes of suicide^1^ are heterogeneous and multilayered. Although the extant literature has identified a wide range of suicide risk factors, the mechanisms through which individuals evaluate worsening circumstances and move toward suicidal thinking remain less understood (Turecki et al. 2019; Zhang et al. 2024).^2^ The prevailing literature on the determinants of suicide is grounded in the expected utility theoretic (EUT) framework introduced by Hamermesh and Soss (1974) (henceforth, *HS*). In the five decades since their contribution, behavioral economists, psychologists, sociologists, and medical scientists have converged in their arguments that suicide does not necessarily grow with age or decrease with socioeconomic status, mainly income—two of the most important predictions of the *HS*'s EUT‐based theory. Appendix A presents a summary of the theoretical prediction of this model.

The utility‐maximizing tradition is not an ideal representation of how individuals evaluate outcomes relative to shifting baselines under distress, how they distort probabilities about the future when new information arrives, and how they update (or fail to update) beliefs after repeated adverse experiences. Smith (2019) argues that the “expected‐utility backbone is empirically fragile and conceptually incomplete as a general theory of suicide.” Some recent research has used laboratory experiments to shed deeper insights into the triggers of suicide. Although a microcosmic view of the determinants of suicide can help design effective mitigation strategies of suicide risk, its wide‐scale implementation is infeasible from the point of view of both cost and logistics, and methodological robustness to a heterogeneous population. An alternative strategy is to design a strong theoretical approach that can mimic the average behavioral traits of individuals' choices of taking risks under uncertainty. These limitations motivate a shift toward decision models that can represent reference dependence, asymmetric loss valuation, and belief distortion. This paper constructs such a theoretical framework in the form of a learning‐driven cumulative prospect theory (CPT) of suicide in the tradition of Tversky and Kahneman (1992)'s seminal contribution. Exploiting a unique household‐level panel data set in South Korea, we then provide strong empirical evidence in support of the theoretical predictions. Recent large‐scale evidence also suggests that suicide‐related outcomes are associated with a broad amalgamation of behavioral and physiological factors, underscoring the need for richer models of individual evaluation and decision‐making under distress (Zhang et al. 2024).

The CPT explains how individuals weigh *losses* more than equivalent gains at uncertain times, and if individuals' learning from the environment matters as the state of their decision transits from ideation and planning, and then to attempting suicide.^3^ The theory supports distinct *fourfold patterns* of choices: individuals tend to be *risk‐averse* for gains and *risk‐seeking* for losses when probabilities are high, but *risk‐seeking* for gains and *risk‐averse* for losses when probabilities are low. The EUT framework, in contrast to CPT, is unable to accommodate how individuals perceive losses and gains relative to a dynamically shifting baseline of well‐being (in their terminology, a *reference point*). In the context of suicidal cognition, the reference point is not fixed at survival but at the perceived intolerability of present suffering. Our contribution differs from recent behavioral prediction work by offering a structural framework in which suicidal ideation (SI) is linked to dynamic reference dependence, loss evaluation, and probability weighting rather than prediction alone (Lalvani et al. 2024).

The prospect function for suicide assigns asymmetric values to perceived gains and losses around a reference point of psychic equilibrium. Near this threshold, perceived “losses” (continued suffering, social isolation, or failure) may loom larger than potential “gains” (hope, recovery, or relational support), thus reflecting an extreme slope in the psychological tipping point (loss domain) of the value function. In this circumstance, the individual may feel that the expected cost of a crisis far exceeds his realized capacity to earn a viable option or even search for it, thus nudging him to make advantageous value‐based choices (Dombrovski and Hallquist 2021). This governing bias and inability to learn may force an individual, even at a younger age and at a higher income level, to ideate, plan, and commit suicide.^4^ Furthermore, nonlinearly weighted future expectations (probability weighting) captures how individuals in despair overweight low‐probability “escape” outcomes (e.g., fantasies of relief through death) while underweighting high‐probability life‐preserving outcomes (e.g., recovery or social reintegration). By recognizing shifting reference points, affective weighting, and nonlinear valuation, a prospect‐based function to understand growth of despair is governed by *bounded rationality* 5 and the asymmetric psychology of suffering and perceived release.

The formalization of SI in a learning‐enabled CPT setting is novel. A growing theoretical literature establishes that learning from experience can generate persistent memory effects in belief formation. Chevillon and Mavroeidis (2017) show that recursive learning rules can produce long‐memory processes even when the underlying shocks are short memory, implying that accumulated negative experiences may interact with current circumstances in ways a purely static model cannot capture. We adopt a tractable short‐memory approximation^6^ consistent with this insight: we define the reference point as the individual's own prior‐period well being, so that gains and losses are evaluated relative to recent personal experience.

In our empirical implementation, we operationalize socioeconomic status using individuals' reported reference or social status. If the reference value changes over time, the gains and losses also become dynamic along with their associated probabilities. An individual's learning can then be derived by estimating the distance function (the difference between the past and present value of one's status and the difference between his and others' value at a point in time). Depending on the (positive/negative) value of this difference, individuals try to learn and adapt their social status. Using such a mechanism, we examine whether CPT‐derived constructs predict this stage,^7^ beyond conventional socioeconomic and clinical covariates (Klonsky et al. 2016; O'Connor and Kirtley 2018).

The rest of the paper is planned as follows. Section 2 provides a thematic overview of the broad literature on suicide. In Section 3, we present a cumulative prospect theoretic setting for SI and discuss various properties vis‐a‐vis existing literature. Section 5 presents empirical evidence from a unique household‐level panel data set. Finally, Section 6 concludes with a detailed discussion of results and policy implications.

## 2 Literature Review

The triggers of suicide are too complex to be identified within a single disciplinary setting. In this section, we present a synoptic overview of the existing literature and, in the process, attempt to minimize repetition of what has already been documented by many notable commentators. We summarize below the notable findings by clustering them into theoretical and empirical literature.

### 2.1 Theoretical Findings

#### 2.1.1 Ideation‐to‐Action and Integrated Motivational‐Volitional Model

Contemporary theory of suicide emphasizes an *ideation‐to‐action* framework, positing that both the emergence of suicidal *ideation* and the progression from *ideation to attempts* are distinct phenomena with partially identifiable predictors (Klonsky et al. 2016). This distinction has direct implications for empirical modeling: variables that are strongly associated with ideation (e.g., depression, hopelessness, broad internalizing symptoms) frequently have limited power to discriminate attempters from ideators, implying that purported prevention policy and robust design of clinical practice require distinct levers at different stages. In this vein, modern theories attempt to separate the *formation of suicidal desire* from the *capacity to act on that desire*. For instance, the Integrated Motivational‐Volitional model formalizes this staging process by arguing that defeat and entrapment give rise to ideation, while *volitional moderators*, such as access to means, exposure to suicidal behavior, acquired capability, planning, impulsivity, and prior behavior, shape progression to suicidal acts (O'Connor and Kirtley 2018). This approach has a deeper connection with the interpersonal theory that highlights perceived burdensomeness and thwarted belongingness as drivers of suicidal desire, and acquired capability as the factor that enables lethal self‐harm (Joiner et al. 2009). These frameworks imply that any economic model of suicide must (i) respect outcome staging (ideation, planning, and attempts) and (ii) allow upstream conditions to operate through both motivational and volitional channels.

#### 2.1.2 EUT‐Based Model of Suicide

*HS* have mathematically formalized elements of both ideation‐to‐action and the integrated motivational–volitional model, known as the expected utility (EUT)‐based economic theory of suicide. The EUT framework treats self‐killing as a (somewhat rational) decision governed by lifetime utility, with distaste for life and expected future utility playing central roles. At the core of this theory is the premise that suicide can be represented as a choice problem in which individuals compare the expected value of continuing life against the alternative of death. Two important predictions emerge from their model: *suicide rates increase with age and fall with higher income*. While this framework remains influential, the empirical record reveals sharp heterogeneity across ages and contexts, as well as strong sensitivity to environments characterized by heightened uncertainty and distress (e.g., macroeconomic shocks) (Lepori et al. 2024). Theoretically, EUT assumes nonlinearly weighted future expectations or linear probability weighting and rational consistency across states of nature.

Marcotte and Hansen (2024) extend the standard utility‐maximizing model by explicitly incorporating a nontrivial probability of survival from a suicide attempt and allowing the attempt itself to affect postattempt utility. In this expanded model, attempts are more likely when, conditional on survival, they raise prospects.^8^ Similarly, Cutler et al. (2001) focus on youth suicide and documents that youth attempts, and completions can follow distinct dynamics from adult patterns. The authors propose that many youth attempts can be interpreted as strategic signaling in conflicts within oneself or with others, and they highlight contagion effects and family structure (notably the rise of divorce) as important correlates of the historical increase in youth suicide (Cutler et al. 2001). These contributions are important because they move beyond a single terminal choice to a setting in which attempts can be nonfatal, socially embedded, and potentially strategic.

A further refinement within economics is that the relevant utility comparison is often inherently social. Using two independent US data sources, Daly et al. (2013) estimate suicide hazards and show that, holding own income constant, suicide risk increases with others' income. This result provides direct evidence that interpersonal comparisons and relative standing enter the suicide‐risk production function (Daly et al. 2013). Related theoretical work argues that suicidal persons choose not only whether, but also when and how to act. By applying search theory to method choice, Whitman (2002) shows that restricting availability of a method can have nonobvious dynamic effects, including the possibility that some individuals may substitute across timing or methods in ways that could, under certain conditions, increase overall suicide risk (Whitman 2002). Collectively, these studies illustrate the power of economic modeling but they also underscore that the empirical object is not merely “completed suicide,” and that social interactions, strategic behavior, and dynamic choice under constraints matter for understanding attempts and transitions.

### 2.2 Empirical Literature

A substantial social‐science literature documents systematic socioeconomic gradients in suicide mortality and related harms across high‐income settings (Lorant et al. 2005; Hiyoshi et al. 2018). Importantly, socioeconomic effects often appear to operate through *relative standing* and perceived social loss rather than through absolute resources alone: relative deprivation in income predicts suicidal ideation/planning and attempt over and above absolute income and material living conditions (Pak and Choung 2020).

Cross‐national evidence—for example, from Western Europe—indicates that, in most settings, greater socioeconomic disadvantage is associated with higher suicide risk, with heterogeneity across countries and generally smaller and less consistent socioeconomic differentials among women (Lorant et al. 2005). Even within welfare states, inequalities can be substantial and may widen following macroeconomic stress. Evidence from Sweden indicates considerable income inequalities in suicide mortality that widened over time, with discussion suggesting that changes in job insecurity and working conditions, including sectoral exposure after recessionary periods, may contribute to the observed pattern (Hiyoshi et al. 2018). Policy evidence is consistent with causal socioeconomic channels, as protective labor‐market policies such as minimum wage increases are associated with reductions in suicide rates among lower‐education groups, particularly during periods of high unemployment (Kaufman et al. 2020).

Taken as a whole, these results support a framework in which social comparison, reference points, and perceived losses under uncertainty play a central rather than a peripheral role. However, despite decades of work, forecasting suicide‐related outcomes remains limited in accuracy, encouraging approaches that move beyond broad correlates toward mechanism‐linked predictors (Ribeiro et al. 2015). The literature increasingly recommends framing suicidal thoughts and behaviors as a dynamic process and explicitly modeling the transition from ideation to action rather than treating all suicide‐related outcomes as a single endpoint. In practice, this means identifying decision‐relevant constructs that (i) plausibly mediate socioeconomic shocks and (ii) discriminate stage transitions, especially the ideation‐to‐attempt margin emphasized by ideation‐to‐action theories (Klonsky et al. 2016; O'Connor and Kirtley 2018).

A crucial refinement for the mechanism is that socioeconomic influences may operate not only through absolute resources but also through *relative* standing and perceived losses versus a salient benchmark. This concept aligns naturally with reference dependence in behavioral economics, where outcomes are evaluated relative to a reference point. In this spirit, Pak and Choung (2020) examine relative deprivation in income as a predictor of suicide‐related outcomes and argues that psychosocial stress may mediate the link between deprivation and health. Empirically, they find that relative deprivation is independently associated with higher odds of SI and suicide planning or attempts, over and above absolute income and material living conditions, with gender heterogeneity depending on the specification of reference groups and deprivation measures (Pak and Choung 2020). The key implication is that a reference‐dependent notion of socioeconomic loss, rather than poverty alone, is relevant for suicide‐related outcomes.

### 2.3 Synthesis

The existing literature supports three relatively well‐established conclusions. *First*, suicide‐related outcomes are most appropriately conceptualized as a multistage process typically encompassing SI, planning, and attempts, in which the relevant risk factors and predictors systematically differ across stages (Klonsky et al. 2016; O'Connor and Kirtley 2018; Joiner et al. 2009). *Second*, socioeconomic disadvantage, income inequality, and relative deprivation exhibit consistent associations with elevated suicide risk, and macroeconomic vulnerability appears to exacerbate these disparities even within high‐income welfare states (Lorant et al. 2005; Hiyoshi et al. 2018; Pak and Choung 2020). *Third*, the decision‐theoretic parameters central to CPT, including loss aversion and probability weighting, have been empirically shown to differ in clinical populations pertinent to suicidal behavior and may offer a mechanistic framework for understanding how individuals subjectively evaluate uncertain future outcomes (Baek et al. 2017; Hadlaczky et al. 2018; Liu et al. 2022; Hagiwara et al. 2022).

However, a key missing link is that these strands are rarely integrated within a unified, longitudinal empirical framework. Much of the socioeconomic literature estimates reduced‐form associations between socioeconomic indicators and suicide outcomes, while much of the computational literature estimates prospect theoretic‐related parameters in clinical samples using laboratory‐style tasks. There is comparatively limited evidence that connects longitudinal socioeconomic trajectories and relative deprivation/reference points to CPT‐consistent valuation and belief constructs, and then to stage‐specific suicidal outcomes in population panels.

Table B.1 in the Appendix B summarizes the two dominant lenses, utility‐based economics and CPT/computational decision models and highlights the missing bridge that motivates our design: socioeconomic trajectories shape reference points and belief updating, and persistent learning failures can govern stage transitions from ideation to attempts. Our contribution is to operationalize this bridge in a longitudinal, stage‐based empirical framework.

**TABLE 1:** Summary statistics.

Statistic	Value
Individuals (estimation sample)	14,552
Person‐year observations	55,884
Survey waves	2018–2023 (6 waves)
SI rate (valid observations)	1.97%
Male share	44.01%
Young share (age ≤35)	17.49%
Mean Cantril Ladder score	6.30 (SD: 1.66)
Mean Ladder | SI =1	4.08 (SD: 1.98)
Mean Ladder | SI =0	6.34 (SD: 1.62)
Ladder coverage per wave	87%–89%
Mean annual well‐being change | SI =1	−0.716
Mean annual well‐being change | SI =0	+0.038
Mean subjective transition probability ${\hat{p}}_{it}$	0.294 (SD: 0.116)

*Note*: Primary cohort (person‐specific baseline, age ≥20). Estimation sample restricted to waves with valid Cantril Ladder, SI response, and one‐period lag (2018–2023). DV missingness of 33.3% reflects survey skip‐pattern design, not selective attrition.

**TABLE 2:** Population CPT parameter estimates versus Tversky and Kahneman (1992) benchmarks.

Parameter	Posterior mean	90% CrI	TK1992	Interpretation
λ (loss aversion)	2.197	[1.33,3.17]	2.25	Losses weighted ≈2.2× gains
α (gain curvature)	0.872	[0.80,0.92]	0.88	Concave value function, gains
β (loss curvature)	0.871	[0.80,0.92]	0.88	Concave value function, losses
γ (prob. weighting, gains)	0.601	[0.42,0.76]	0.69	Stronger prob. distortion
δ (prob. weighting, losses)	0.618	[0.44,0.78]	0.69	Stronger prob. distortion
$\beta_{\mathrm{cpt}}$	−1.010	[−1.63,−0.60]	—	Lower CPT value ⇒ higher SI
Intercept κ	−4.22	[−4.47,−3.98]	—	Baseline log‐odds of SI
Max $\hat{R}$	1.0025			
Min ESS (bulk)	2071			
Divergences	0			

*Note*: Hierarchical Stan model, 4 chains × 1000 sampling draws. Prior on $\beta_{\mathrm{cpt}}$: N(0,2) (noninformative, no directional assumption). Population hyperpriors centered on TK1992 benchmarks (Tversky and Kahneman 1992). A total of 90% credible intervals reported.

**TABLE 3:** GEE panel estimates lagged CPT value and suicidal ideation.

Variable	Coef.	SE	Wald	p‐Value	OR	95% CI (OR)
Intercept	−3.930	0.082	2289.4	<0.001	—	—
CPT value (lagged)	−0.204	0.059	12.14	0.0005	0.815	[0.727,0.915]
COVID‐19 indicator (yr 2020)	−0.027	0.108	0.061	0.805	0.974	[0.788,1.203]

*Note*: GEE with exchangeable working correlation structure. Cluster‐robust standard errors at the individual level (h_pid). N=41,331 person‐year observations, 14,552 individuals. CPT value lagged one period: well‐being trajectory at t−1 predicts SI at t. OR $=\exp(\hat{\beta })$.

**TABLE 4:** Pearson correlations between individual CPT parameters and suicidal ideation rates.

Parameter	Domain	r	p‐Value	Significance
λ (loss aversion)	Loss	+0.629	<0.001	***
δ (prob. weighting, losses)	Loss	+0.554	<0.001	***
β (loss curvature)	Loss	+0.146	<0.001	***
γ (prob. weighting, gains)	Gain	−0.072	0.022	*
α (gain curvature)	Gain	+0.030	0.337	—

*Note*: Pearson correlations between individual posterior mean CPT parameters (N=1000 subsampled individuals) and individual‐level mean SI rates. Significance: *** p<0.001, * p<0.05.

**TABLE 5:** Average marginal effects of lagged CPT value on Pr(SI=1).

Subgroup	N (obs)	N (SI)	AME	SE	p‐Value	95% CI
Full sample	41,331	815	−0.0061	0.0009	<0.001	[−0.0080,−0.0043]
Male	17,118	278	−0.0056	0.0013	<0.001	[−0.0082,−0.0029]
Female	24,213	537	−0.0065	0.0013	<0.001	[−0.0090,−0.0039]
Young (≤35)	4629	98	−0.0095	0.0030	0.002	[−0.0153,−0.0036]
Older (>35)	36,702	717	−0.0057	0.0010	<0.001	[−0.0076,−0.0037]
Female × Young	2627	73	−0.0110	0.0046	0.016	[−0.0200,−0.0021]
Male × Young	2002	25	−0.0075	0.0035	0.030	[−0.0143,−0.0007]
Female × Older	21,586	464	−0.0060	0.0013	<0.001	[−0.0086,−0.0033]
Male × Older	15,116	253	−0.0052	0.0015	<0.001	[−0.0080,−0.0024]

*Note*: AME computed from GEE logit (exchangeable correlation, cluster‐robust SE at individual level). All AMEs are negative: higher lagged CPT value reduces SI probability. Young defined as age ≤35.

**TABLE 6:** Subsample GEE estimates during pandemic.

Sample	$N_{\text{obs}}$	$N_{\text{ind}}$	SI%	OR	95% CI	p
*Panel A: COVID‐period splits*						
Full sample (baseline)	41,331	12,722	1.97	0.815	[0.727, 0.915]	<0.001
Non‐COVID (2018–2019, 2022–2023)	25,534	12,382	1.97	0.806	[0.701, 0.927]	0.002
COVID only (2020–2021)	15,797	8816	1.98	0.765	[0.632, 0.925]	0.006
*Panel B: New‐onset restriction*						
New‐onset (${\text{SI}}_{t-1}=0$)	28,042	9212	1.62	0.795	[0.682, 0.928]	0.004

*Note*: All specifications use GEE with exchangeable working correlation structure and cluster‐robust standard errors at the individual level. Dependent variable: suicidal ideation (${\text{SI}}_{it}\in \left\{0,1\right\}$). Key regressor: lagged CPT value ($V_{i,t-1}$). Year fixed effects included in all specifications. OR $=\exp(\hat{\beta })$. Panel A reports separate GEE estimates for COVID‐period waves (2020–2021) and non‐COVID waves; the OR stability ratio is 0.765/0.806=0.949, indicating near‐identical effect magnitudes across periods. Panel B restricts to person‐years with ${\text{SI}}_{i,t-1}=0$, eliminating the ideation‐persistence channel through which prior suicidal ideation could have depressed the lagged CPT value.

**TABLE 7:** CPT × COVID interaction test.

Specification	OR	95% CI	p
Baseline GEE (no interaction)	0.815	[0.727, 0.915]	<0.001
*Interaction model* (CPT×COVID):			
CPT value ‐ non‐COVID (baseline)	0.808	[0.704, 0.927]	0.002
CPT value ‐ COVID period	0.826	[0.677, 1.008]	0.060
Interaction term (CPT × COVID)	1.022	[0.804, 1.300]	0.858

*Note*: GEE with exchangeable working correlation structure and cluster‐robust standard errors at the individual level. N=41,331 person‐years; $N_{\text{ind}}=12,722$. Dependent variable: ${\text{SI}}_{it}$. The interaction model includes lagged CPT value, a precomputed CPT × COVID interaction term, and year fixed effects; the stand‐alone COVID dummy is excluded to avoid perfect collinearity with year fixed effects. The CPT coefficient in the interaction model identifies the effect in non‐COVID years; the COVID‐period effect is recovered as the sum of the CPT and interaction coefficients with standard errors computed via the delta method. A significant positive interaction (OR >1, p<0.05) would support reverse causation; the observed interaction OR of 1.022 is statistically null (p=0.858), inconsistent with reverse causation as the primary driver of the CPT–SI association.

## 3 Prospect Theory Design of Suicide

We noted in Section 1 that a prospect‐theoretic formalization can offer a behavioral microfoundation for how socioeconomic and psychological states are evaluated under distress. At a simplified level, an individual facing severe distress may evaluate uncertain future states involving continued suffering, possible recovery, social consequences, and perceived relief. The framework is not intended to describe a conscious calculation between life and death. Instead, it represents how perceived gains and losses around a reference point may shape the subjective evaluation of future well‐being under uncertainty. At a simplified decision‐theoretic level, an individual facing severe distress evaluates two broad probabilistic outcomes: (i) *continue to live* despite persistent suffering (subjective losses), but there is a possibility of life improvements (subjective gains), and (ii) *attempt suicide* because he expects a complete relief from continuous pain (subjective gain), but death (or self‐harm) impacts others (subjective losses). Both options (i.e., continue to live despite suffering, and attempting suicide) involve uncertain outcomes, because the individual would not know with certainty if there will be an improvement in his life,^9^ or if his suicide (attempt) can actually be the solution to his suffering and to be of indifferent consequence to others.

### 3.1 Theory

At the core of CPT are two psychological functions: a *subjective value function* U for an outcome *x*, written *U(x)*, and a *subjective probability weighting function* W(p). The latter is typically inverse *S*‐shaped, overweighting small probabilities and underweighting moderate to large ones. This captures people's tendency to assign excessive decision weight to rare events and to downplay common ones. Positive future outcomes may lose subjective value, while relief from pain may become disproportionately valued. Similarly, individuals may overweight the probability that suffering will persist and underweight the probability of recovery (or a smooth life course and the risk of death or irreversible harm).

While static CPT assumes a fixed reference point, in our setting the relevant cognitive process is intertemporal: expectations evolve as individuals internalize past shocks, accumulate memories, and reassess their position relative to peers. Probability weighting does not imply that people objectively misestimate probabilities; rather, given a perceived or inferred probability, the associated decision weight may be nonlinear. Probability overestimation instead concerns biased belief formation about how likely an event is (e.g., via availability bias or response formats that discourage very small numbers). In our empirical application, subjective probabilities are inferred from observed well‐being transitions; CPT weighting is then applied to these inferred probabilities. Belief construction and probability weighting are accordingly conceptually distinct.

To formalize presentation, consider an individual facing uncertain future outcomes. Assume that these outcomes are captured by the individual's *subjective well‐being changes* x relative to a personal reference point r. For instance, the individual's personal reference point can be a point where the individual feels to be at break‐even in terms of well‐being or has a better sense of happiness (leading to the continuation of life). Changes in subjective well‐being can occur following changes in circumstances in life (which can be influenced by the broader socioeconomic environment, among others). Therefore, *r* can be greater than *x*, and as long as *x* conforms to the expectation of *r*, the individual may feel happy about life to continue, and hopefully make it better over time. Let us denote $\left(p_{it}\right)$ (model‐implied) probability of future well‐being improvement, while $\left(w\left(p_{it}\right)\right)$ denotes the (CPT) decision weight attached to that probability for individual i at time *t*. Denote $W_{it}$ as the subjective well‐being^10^ of individual i at time t. Consistent with reference‐dependent preferences, individuals evaluate their current well‐being relative to their recent experience. The individual reference point is defined as

(1) $$r_{it}=W_{i,t-1}.$$

Changes in well‐being ($x_{it}$) (i.e., the difference between today's well‐being, $W_{it}$ and yesterday's (i.e., the reference point: $W_{i,t-1}$) is

(2) $$x_{it}=W_{it}-W_{i,t-1},$$

where positive values of $x_{it}$ represent upward movements on the Cantril Ladder (gains), while negative values represent downward movements on the same ladder scale (losses). This formulation captures an important trait: individuals evaluate their present condition relative to their own past experience rather than to a fixed external benchmark. Therefore, changes in well‐being ($x_{it}$), can be characterized behaviorally and quantitatively by designing a prospect value function:

(3) $$v\left(x_{it}\right)=\left\{\begin{array}{cc} x_{it}^{\alpha }, & x_{it}\geq 0\\ -\lambda {\left(-x_{it}\right)}^{\beta }, & x_{it}<0, \end{array}\right.$$

where α and β capture diminishing sensitivity for gains and losses, respectively, and λ>1 represents loss aversion. Loss aversion implies that declines in well‐being have a stronger psychological impact than equivalent improvements. Indeed, individuals often lay more importance on bad shocks, which influences their subsequent choices more than if they had experienced equivalent gains.^11^

#### 3.1.1 Subjective Expectations of Future Well‐Being

CPT also requires a representation of individuals' beliefs about future outcomes. We construct subjective probabilities of future well‐being changes using a Markov transition framework. Denote by $s_{it}$ the discrete well‐being state associated with a predefined score (in our case, the Cantril ladder score). Individual well‐being trajectories generate transitions between states over time. For each individual i, we estimate the probability of transitioning from state s to state $s^{\prime }$:

(4) $$P_{i}\left(s,s^{\prime }\right)=Pr\left(s_{i,t+1}=s^{\prime }|s_{it}=s\right).$$

Because individual observation histories are limited, transition probabilities are estimated using a Dirichlet–multinomial posterior that partially pools individual transitions toward the population distribution. The subjective probability that well‐being improves in the next period is defined as

(5) $$p_{it}=\sum_{s^{\prime }>s_{it}}P_{i}\left(s_{it},s^{\prime }\right).$$

This probability captures each individual's belief about the likelihood of future improvement in well‐being. Combining outcome changes $x_{it}$ and subjective probabilities $p_{it}$, the *CPT valuation* of individual prospects is defined as

For *gains*:

(6) $$V_{it}=x_{it}^{\alpha_{i}}w^{+}\left(p_{it};\gamma_{i}\right)$$

and for *losses*:

(7) $$V_{it}=-\lambda_{i}{\left|x_{it}\right|}^{\beta_{i}}w^{-}\left(1-p_{it};\delta_{i}\right).$$

In this equation, $\lambda_{i}$, $\alpha_{i}$, $\beta_{i}$, $\gamma_{i}$, and $\delta_{i}$ are individual‐specific CPT parameters governing loss aversion, value curvature, and probability weighting. Combining well‐being changes and subjective probabilities, the behavioral valuation of future prospects for the individual *i* at time *t* is given by

(8) $$V_{it}=w\left(p_{it}\right)\cdot v\left(x_{it}\right),$$

where w(p) is the probability weighting function, which is expressed as

(9) $$w\left(p\right)=\frac{p^{\gamma }}{{\left(p^{\gamma }+{\left(1-p\right)}^{\gamma }\right)}^{1/\gamma }}.$$

The parameter γ∈(0,1] governs the curvature of the weighting function. Values of γ<1 generate the characteristic inverse‐*S* shape, implying overweighting of small probabilities and underweighting of large probabilities.

The prospect‐theoretic model generates several empirically testable predictions: (i) SI should be more likely when individuals experience declines in subjective well‐being (loss‐domain asymmetry). (ii) Behavioral heterogeneity in suicide risk should be driven primarily by loss‐side parameters of the value function. (iii) Changes in well‐being should be more predictive of SI than absolute well‐being levels, and (iv) individuals experiencing negative shocks should assign lower subjective probabilities to future improvement. The empirical analysis presented in the following section evaluates these predictions using longitudinal household panel data.

## 4 Data and Estimation Strategy

### 4.1 Data and Measurement of Variables

**Data Selection**: Obtaining individual‐level suicide data (ideation, survival) is difficult. To demonstrate the application of our CPT design of suicide, it is necessary to use individual‐level data, observed over time, alongside various socioeconomic indicators. To serve our purpose, we have used the KoWePS, a nationally representative longitudinal survey administered jointly by the Korea Institute for Health and Social Affairs and Seoul National University.^12^ KoWePS tracks a stratified probability sample of Korean households across a comprehensive range of socioeconomic, mental health, administrative, and contextual dimensions, constituting one of the most detailed longitudinal data infrastructures available for studying subjective well‐being and its behavioral consequences in East Asia. The raw panel encompasses *305,227 person‐year observations* from 31,988 unique individuals surveyed annually between 2005 and 2023, spanning 2798 variables.^13^ The raw SI rate is 2.51%.

**Well‐Being Measure**: A key innovation introduced in this study is the adoption of the *Cantril Self‐Anchoring Scale* (Cantril 1965), hereafter the Cantril Ladder, as the primary measure of subjective economic well‐being (SEW). The Cantril Ladder asks respondents to place themselves on a scale from 0 (worst possible life) to 10 (best possible life), eliciting a direct, integrative, self‐referential evaluation of life position. This measure is theoretically optimal for a CPT application for a fundamental reason: CPT's value function operates on *perceived* rather than objective outcomes, and the Cantril Ladder is the most direct available elicitation of that perception. Unlike composite indices constructed from objective economic components, the Ladder collapses the individual's multidimensional life evaluation into a single self‐referential judgment, precisely the latent state $W_{it}$ that CPT's reference‐dependent framework requires.

To validate this choice empirically, we computed the within‐person (demeaned) correlation between the Cantril Ladder and log household disposable income across the 2018–2021 overlap period. The within‐person correlation is 0.063, statistically negligible, while the cross‐sectional level correlation is 0.350. This sharp divergence reveals a fundamental construct distinction: income tracks *who you are* in the cross section your socioeconomic rank but tells us almost nothing about *how you are doing* relative to your own past trajectory. Since CPT's reference point is inherently intertemporal and self‐referential (Helson 1964; Tversky and Kahneman 1992), and since it is welfare *dynamics* rather than welfare *levels* that we seek to explain, the Cantril Ladder is the unambiguous lead specification. This finding resonates with the Easterlin Paradox (Easterlin 1974): rising aggregate incomes do not reliably translate into rising subjective well‐being, precisely because life satisfaction is anchored to personal reference points rather than absolute material standards.

The Cantril Ladder was first administered in 2017. Note that our CPT framework requires a one‐period lag to construct the reference point, the effective estimation window begins in 2018. The primary cohort is constructed using a person‐specific baseline rule: each individual enters at the first survey wave with a valid SI response, provided they are aged 20 or above. After eligibility restrictions, the primary cohort comprises 19,995 individuals and 225,791 person‐year observations. After requiring jointly valid ladder scores, SI responses, and lag availability, the CPT estimation data set contains 14,552 individuals and 55,884 person‐year observations across six waves (2018–2023). The validated SI rate in the estimation sample is 1.97%. Sample demographics are 44% male and 82.5% aged above 35, consistent with KoWePS panel composition. The 33% missingness in the SI variable reflects a survey skip‐pattern design rather than random attrition. Summary statistics are presented Table 1.

#### Variables

The empirical framework proceeds through four sequential steps: (i) measurement of SI; (ii) construction of the subjective well‐being measure; (iii) definition of the reference point and subjective transition probabilities via a Bayesian Markov framework; and (iv) CPT valuation and hierarchical Bayesian estimation. Each step is motivated by a specific theoretical requirement of the CPT framework, and the architecture is deliberately integrated so that no component is arbitrary.

**SI—Outcome Measurement**: SI is measured using the KoWePS item, recording whether respondents seriously considered suicide during the past 12 months, coded 1 for affirmative, 0 otherwise. This item captures the threshold at which psychological distress has crystallized into self‐directed cognition and has been administered consistently across all waves since 2005. Throughout the analysis, SI is treated as a binary behavioral indicator of accumulated psychological burden associated with reference‐dependent socioeconomic evaluation and subjective uncertainty about the future.

We use the Cantril Ladder as the SEW Measure to construct our reference point. The Cantril Self‐Anchoring Scale (Cantril 1965) operationalizes subjective well‐being as a holistic self‐assessment: respondents identify the rung on a 0–10 ladder that best represents their life, where the anchors are self‐defined. This self‐anchoring property makes the Cantril Ladder a direct elicitation of the individual's own reference frame, precisely what CPT's value function requires. The main alternative to this reference‐dependent specification is an absolute‐level model in which SI is related to current Cantril Ladder level, income, health status, or other contemporaneous socioeconomic indicators. Our approach differs by asking whether movements relative to one's own prior well‐being carry additional behavioral information. Thus, the comparison is not between subjective and objective well‐being alone, but between absolute levels and reference‐dependent changes. In the estimation sample, the Ladder has a mean of 6.30 and a standard deviation of 1.66, spanning the full valid range. Coverage within the analysis sample is high and improving, with 9000–11,700 jointly valid ladder‐and‐SI observations per wave. The raw Ladder gap between the SI = 1 group (4.08) and SI = 0 group (6.34) is 2.26 points, approximately 1.4 standard deviations, and constitutes the most striking descriptive finding in the data.

**Reference Point**: As noted in Section 3, we follow Adaptation Level Theory (Helson 1964) to define the reference point as each individual's own prior‐period well‐being, $x_{it}\;=\;W_{it}-W_{i,t-1}$, where $W_{it}$ denotes individual *i*'s Cantril Ladder score in year t. The Easterlin Paradox is in part explained by precisely this mechanism: as average conditions improve, reference points adapt upward, leaving subjective well‐being unchanged (Easterlin 1974; Clark et al. 2008). In the final sample, $x_{it}$ has a median of zero, a mean of +0.023, and an interquartile range of [−1,+1]. The mean outcome among SI =1 observations is −0.716, compared with +0.038 among the rest =0 observations those who experience ideation are not only situated at lower rungs of the ladder but are, on average, descending. Figure 6 illustrates the distribution of $x_{it}$.

**FIGURE 1:**
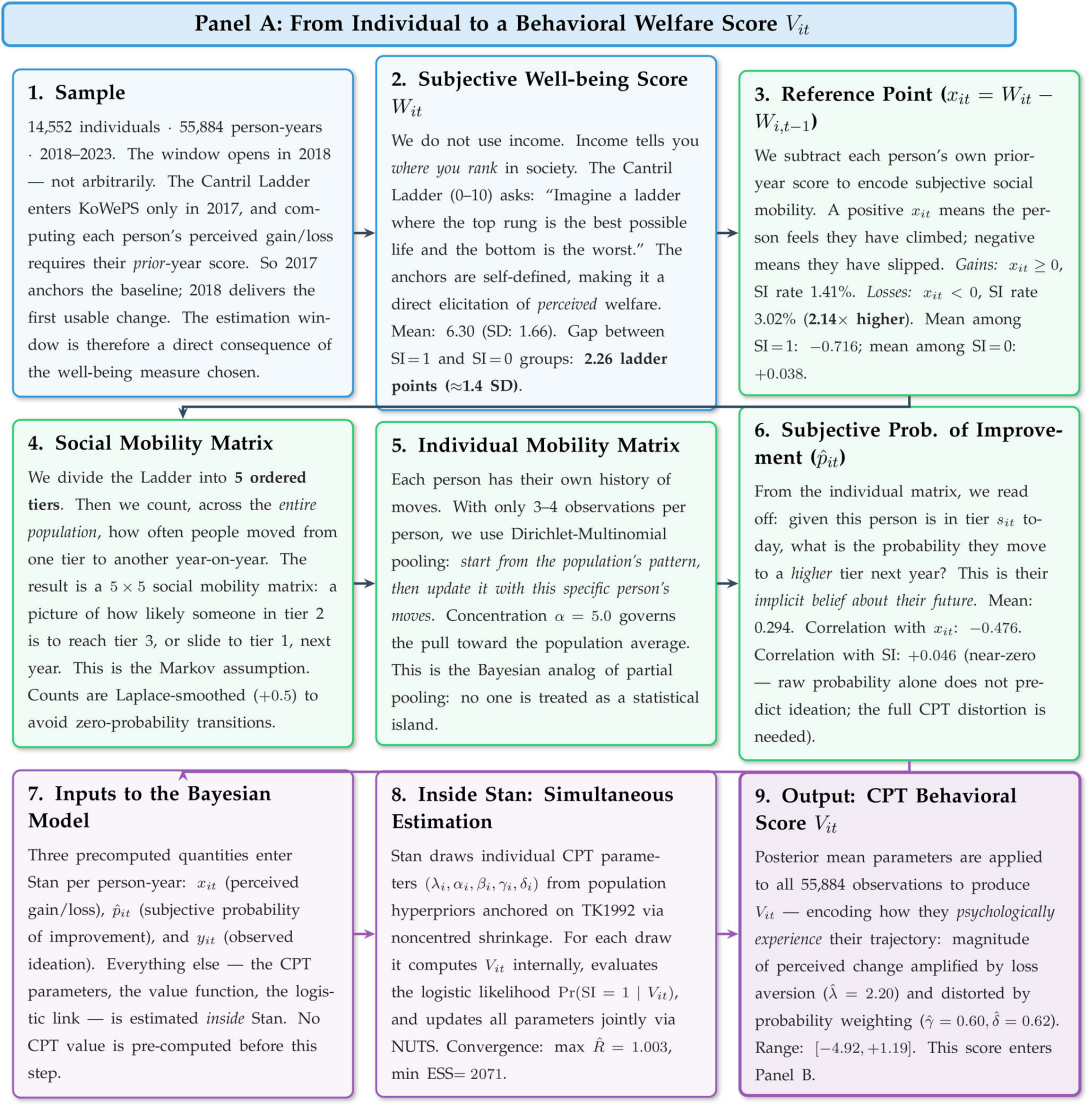
Methodological flowchart Panel A: From survey data to behavioral welfare score $V_{it}$. The workflow proceeds from raw well‐being elicitation and reference point construction (Row 1) to the generation of subjective social mobility beliefs via Dirichlet‐Multinomial pooling (Row 2), concluding with the simultaneous hierarchical Bayesian estimation of individual CPT parameters in Stan (Row 3).

**FIGURE 2:**
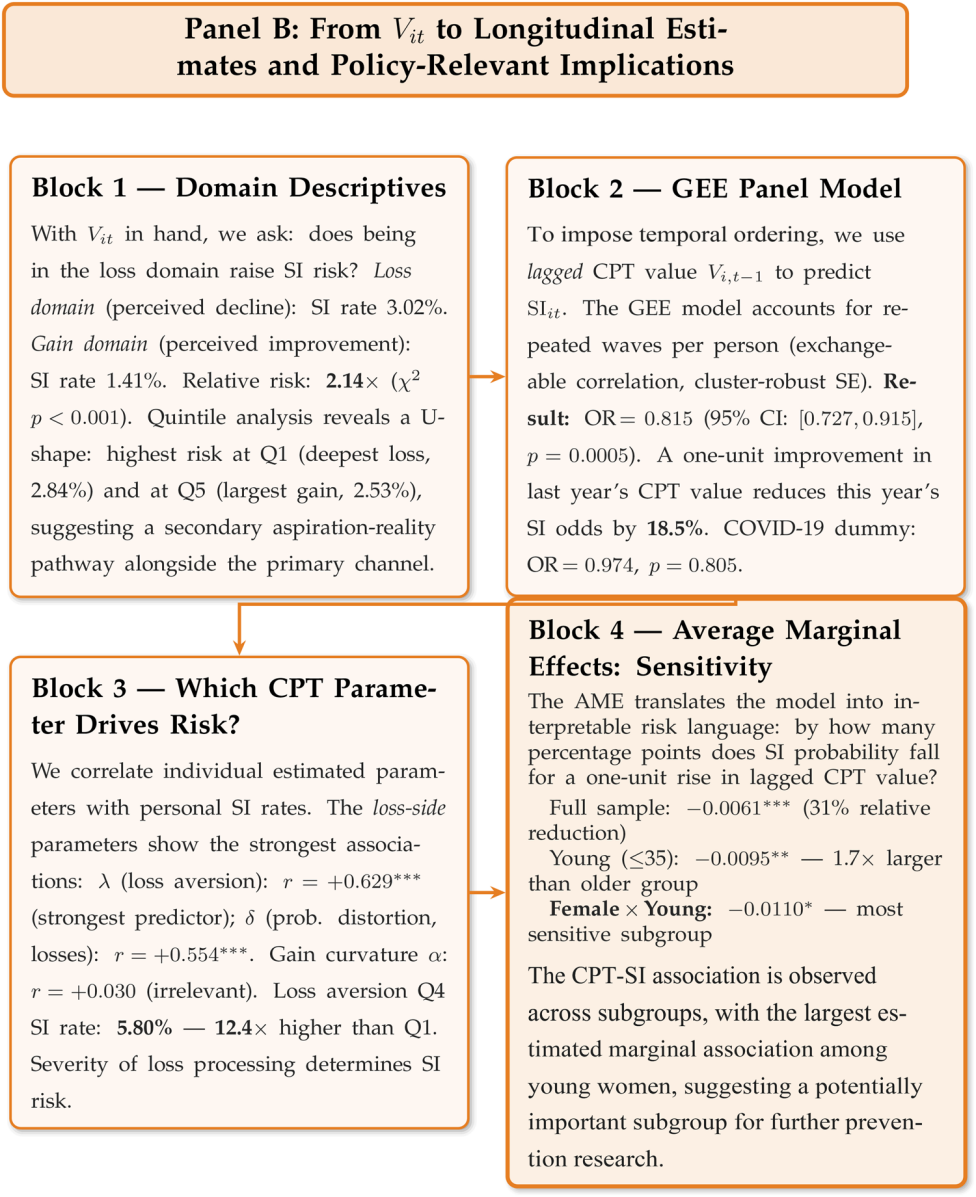
Methodological flowchart Panel B: Causal estimates and policy‐relevant results. This panel details the translation of behavioral scores into longitudinal risk estimates. The analysis moves from descriptive risk concentration (Block 1) to causal panel modeling using GEE (Block 2), identifies loss aversion as the primary psychological driver of ideation (Block 3), and concludes with subgroup‐specific Average Marginal Effects for targeted policy intervention (Block 4).

**FIGURE 3:**
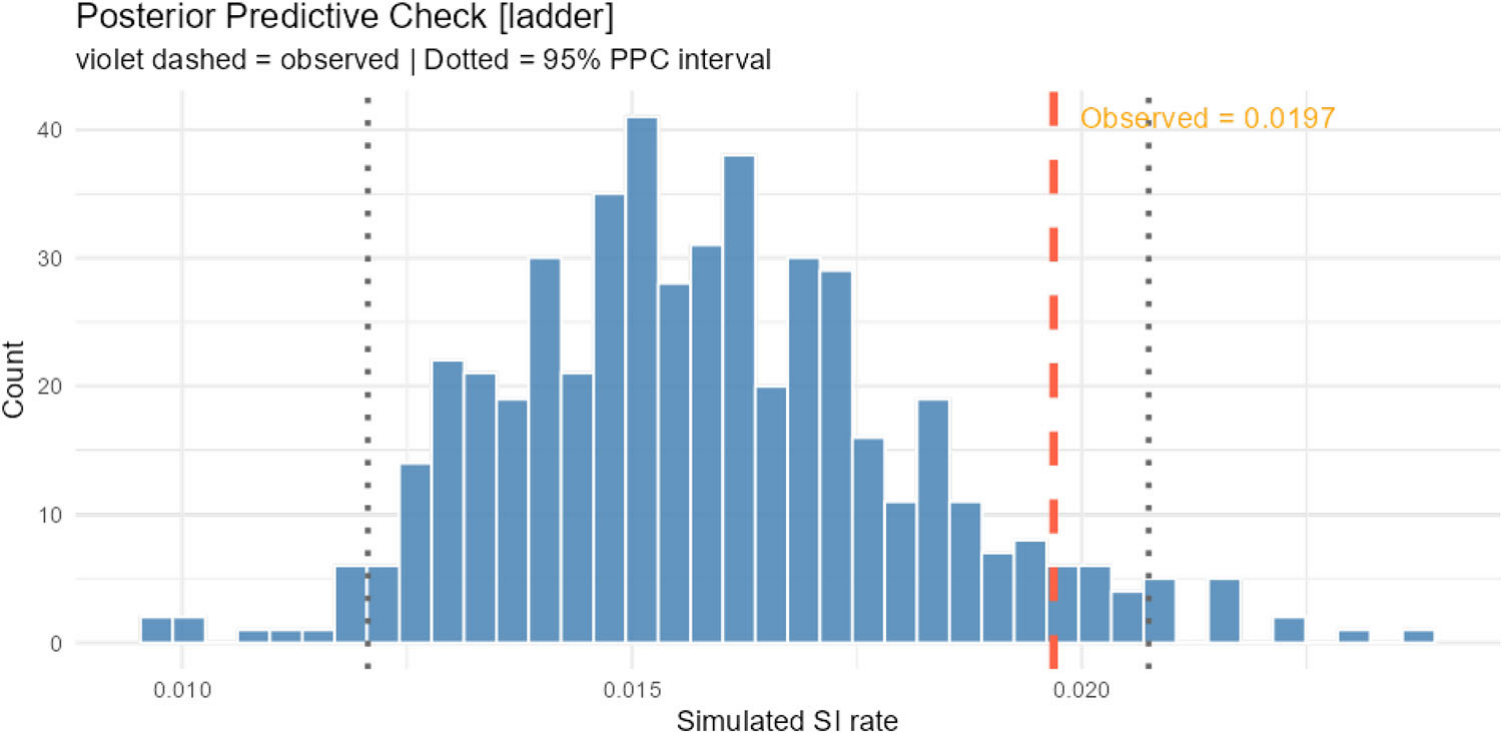
Posterior predictive check (PPC): Model‐simulated vs. Observed suicidal ideation rates. *Note*: This graph represents the distribution of population‐level suicidal ideation (SI) rates generated from 400 posterior predictive draws of the hierarchical CPT‐logistic model. Simulations were carried out for all 55,884 person‐year observations using posterior mean individual parameters $\left({\hat{\lambda }}_{i},{\hat{\alpha }}_{i},{\hat{\beta }}_{i},{\hat{\gamma }}_{i},{\hat{\delta }}_{i}\right)$ alongside the estimated logistic intercept and ${\hat{\beta }}_{\text{CPT}}$. The red dashed vertical line indicates the observed sample SI rate of 1.97%, which falls at approximately the 82nd percentile of the posterior predictive distribution (${\text{mean}}_{\text{ppc}}\approx 0.016$, ${\text{SD}}_{\text{ppc}}\approx 0.002$). The significant overlap between the observed rate and the predictive distribution density confirms the model's capacity to replicate aggregate SI prevalence, demonstrating adequate global fit with a modest upward bias in the predictive mean.

**FIGURE 4:**
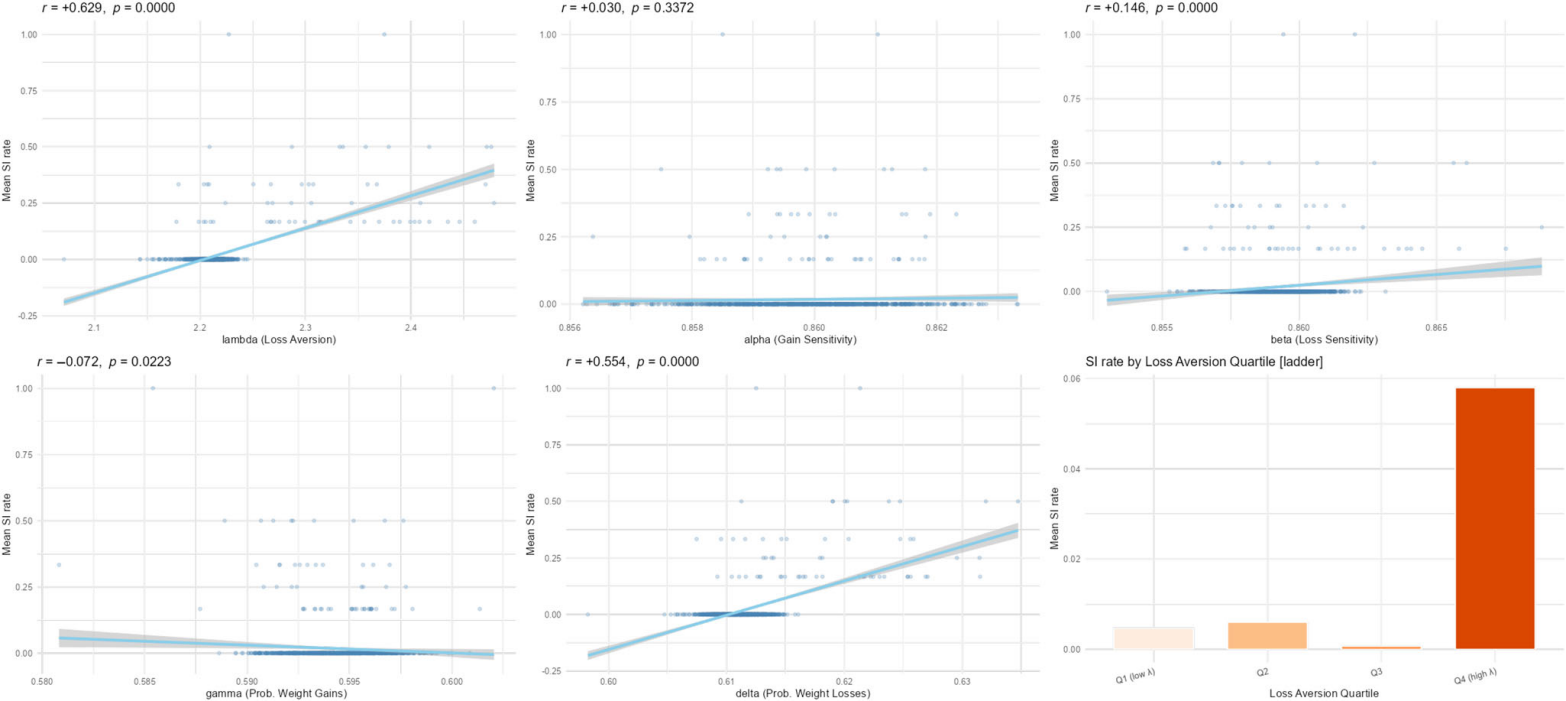
Individual CPT parameter estimates and suicidal ideation rates. *Note*: Each panel plots individual posterior mean CPT parameters against mean SI rates (N=1000 subsampled individuals). Loss‐side parameters (λ, δ, β) show significant positive associations with SI risk; gain‐side parameters (α, γ) are orthogonal. Bottom‐right panel shows mean SI rate by loss aversion quartile; excess risk concentrates discontinuously in Q4 (high $\hat{\lambda }$). Narrow x‐axis ranges reflect hierarchical shrinkage toward population hyperpriors.

**FIGURE 5:**
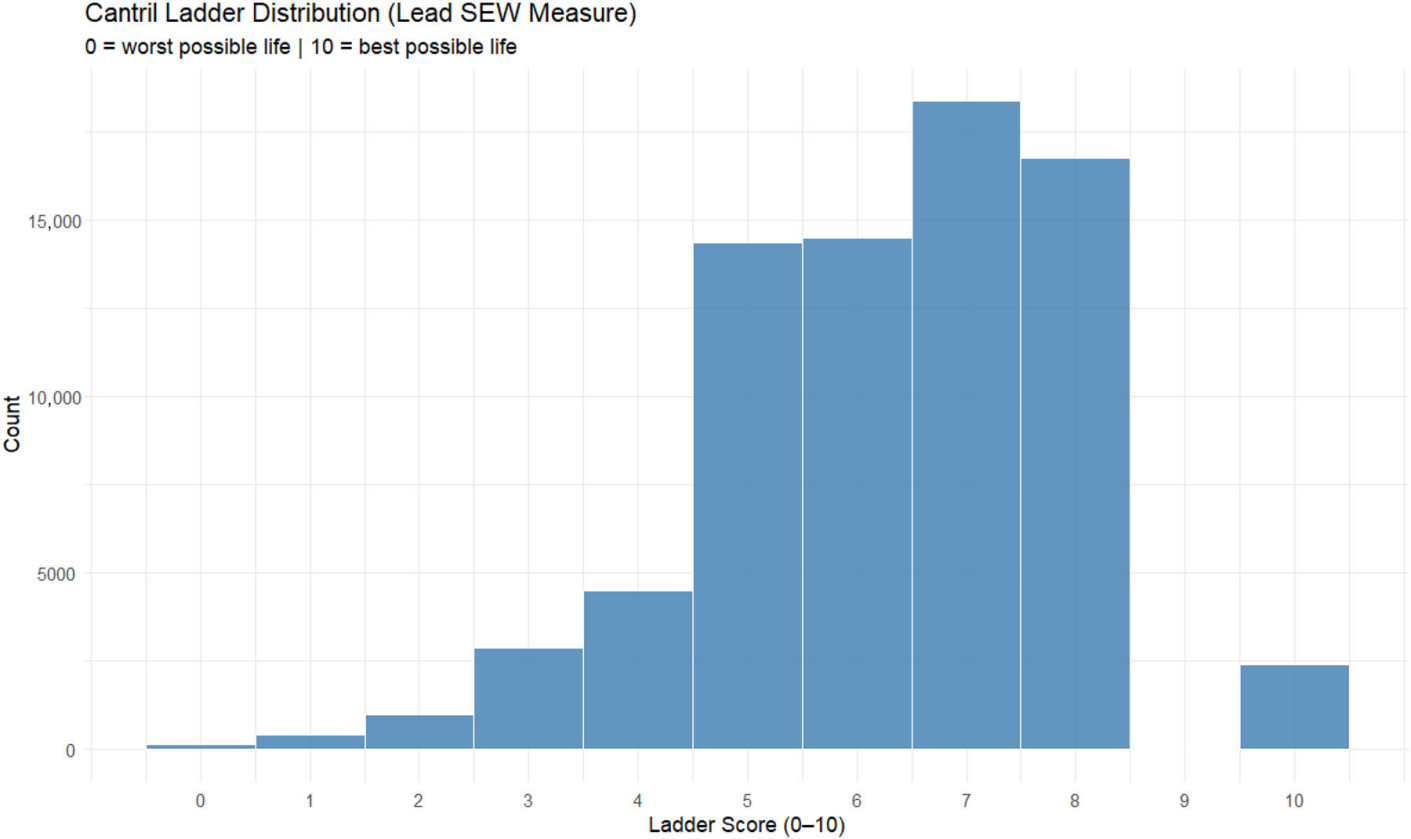
Cantril Ladder distribution (Lead SEW Measure, 2018–2023). *Note*: The scale ranges from 0 (worst possible life) to 10 (best possible life). The distribution is left‐skewed with modal responses at 7–8, consistent with life satisfaction distributions. The thin left tail (scores 0–3) represents the at‐risk population.

**FIGURE 6:**
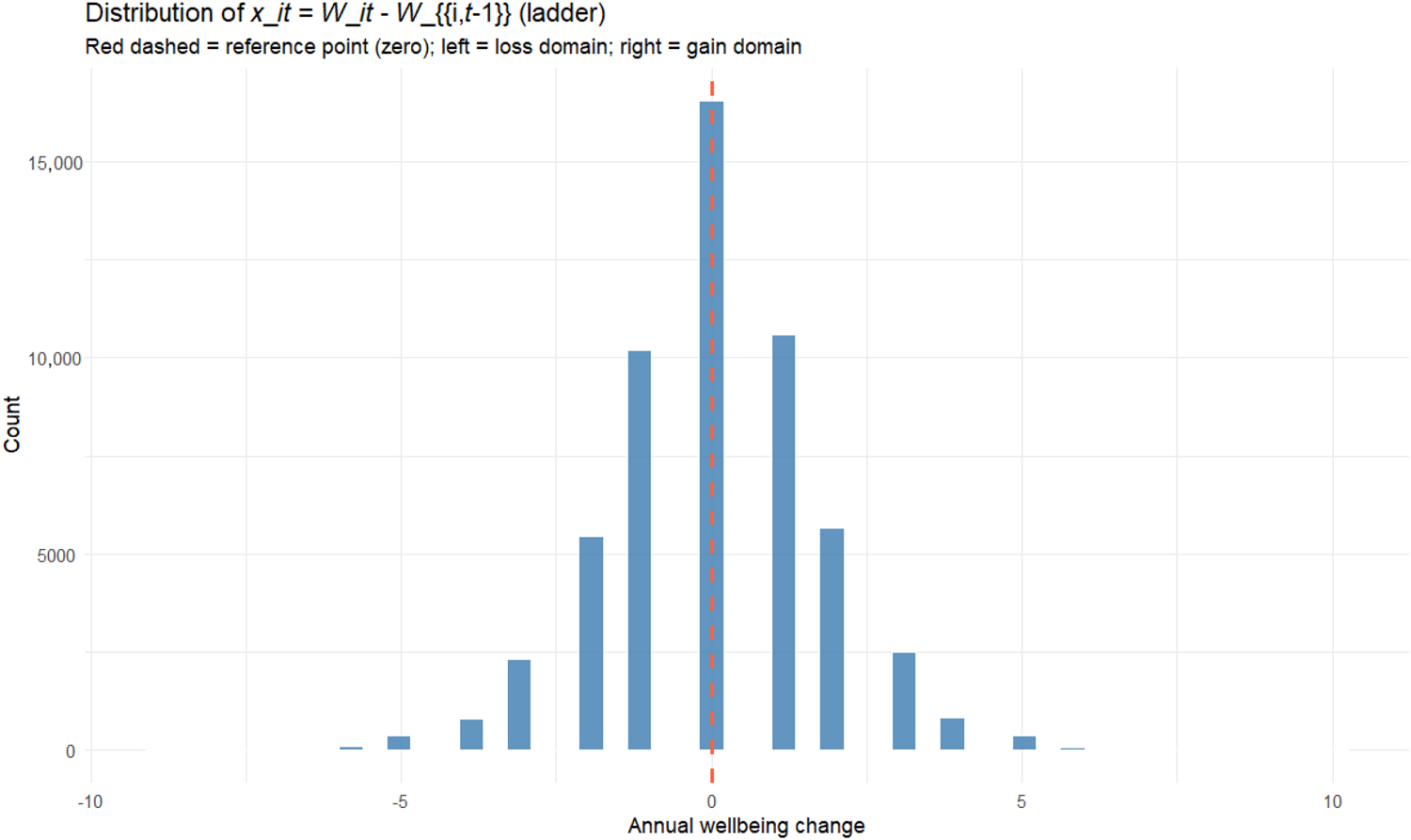
Distribution of $x_{it}=W_{it}-W_{i,t-1}$ (Annual well‐being change). *Note*: The red dashed line marks the reference point (zero). Observations to the left enter the CPT loss domain; observations to the right enter the gain domain. The distribution is approximately symmetric around zero (mean: +0.023; IQR: [−1,+1]), consistent with the integer‐valued Cantril Ladder.

The prospect framing we have presented does not imply *conscious deliberation*, but formalizes the *implicit valuation processes* through which suicide may be perceived as a viable (not a rational) option. What is important here is that individuals evaluate suicidal outcomes relative to a *reference point*, **r,** rather than in absolute terms. The reference point may reflect the expected quality of life, social norms, past peak experiences, or adaptive aspirations. Several behavioral findings in the literature align with decision perspectives, arguing that suicide is linked to impairments in valuation, reward processing, and learning dynamics, which can alter how individuals forecast future states and respond to feedback (Dombrovski and Hallquist 2017; Dombrovski et al. 2013).

Recent work shows that a short reward/aversion judgment task combined with limited demographic and mental‐health measures can predict distinct subjective well‐being outcomes (passive ideation, active ideation, planning, and planning for safety), with peak performance reported for predicting planning and with distinct feature importance patterns across outcomes (Lalvani et al. 2024). This result is methodologically important for our purpose as it suggests that (i) quantitative judgment variables encode meaningful stage‐specific signals and (ii) scalable, nonclinical measurement of decision primitives may be feasible.

### 4.2 Estimation Strategy

Because our goal is to understand how people form expectations about their future well‐being and how these expectations, together with recent changes in well‐being, relate to SI, our analysis follows three steps of estimation. The first involves tracking changes in well‐being over time. Each year, respondents report their well‐being using the discretized Cantril Ladder,^14^ a scale ranging from 0 (worst possible life) to 10 (best possible life). Rather than focusing only on a person's current score, we examine whether their well‐being has improved or worsened compared with the previous year. To achieve this, we compare each person's current well‐being score with their own score from the preceding survey wave. This allows us to identify whether an individual feels that their situation is getting better, staying the same, or deteriorating over time. The key idea is that people often judge their circumstances relative to where they were before, rather than against an objective standard. A decline from one's previous level of well‐being may therefore have important psychological consequences even if the person's absolute level of well‐being remains moderate.

Our second step involves estimating expectations about the future. Indeed, the theory developed in this paper assumes that people evaluate not only their current situation but also their beliefs about what may happen next. To estimate these beliefs, we examine how individuals' well‐being tends to change over time. We first group well‐being scores into five broad categories, ranging from relatively low to relatively high well‐being. We then observe how people move between these categories from one year to the next. For example, some people who are currently in a low well‐being category move to a higher category the following year, while others remain where they are or experience further declines.

To formalize, let $s_{it}\in \left\{1,2,3,4,5\right\}$ denote individual *i*'s state (in well‐being) in year t. For each individual, we observe a sequence of state transitions $\left(s_{it},s_{i,t+1}\right)$ (e.g., from poor to very high well‐being state or a reversal over time). We can then estimate a personal K×K transition matrix. Using these observed patterns, we estimate the probability that each individual will experience an improvement in well‐being in the future. Because most people are observed for only a limited number of years, there is often insufficient information to estimate these probabilities reliably for each person separately. We therefore combine information from an individual's own history with information from the wider population. This approach allows us to create a more stable estimate of each person's perceived likelihood of future improvement while still preserving meaningful individual differences.

Because individuals are observed for only six survey waves, we use a Bayesian partial‐pooling approach that combines information from each person's own well‐being history with information from the wider population. This produces more reliable estimates when individual data are limited, while still allowing people to differ from the population average. As a result, estimated probabilities reflect both individual experience and broader population patterns. We apply a Dirichlet‐Multinomial posterior with partial pooling toward the population‐level matrix. In this mechanic, we treat the concentration parameters of the individual groups as random draws from a shared population‐level distribution. This approach pulls (shrinks) group‐specific probabilities toward the population matrix while retaining the ability to learn group‐specific deviations, which is the case in our data. Accordingly,

(10) $$P_{i}\left[s,\cdot \right]\;∼\;\mathrm{Dir}\;\left(\alpha \;P_{\mathrm{pop}}\left[s,\cdot \right]+n_{i}\left[s,\cdot \right]\right),$$

where α=5.0 is the concentration parameter governing the strength of shrinkage toward the population matrix $P_{\mathrm{pop}}$, and $n_{i}\left[s,s^{\prime }\right]$ counts individual *i*'s observed transitions from state s to state $s^{\prime }$. The population matrix $P_{\mathrm{pop}}$ is estimated using Laplace‐smoothed (+0.5) aggregate transition counts. This Dirichlet‐Multinomial construction provides Bayesian‐coherent individual‐level estimates that borrow strength from the population while respecting individual trajectories where data are sufficient.^15^ Indeed, as more individual information becomes available, the estimates rely increasingly on that person's own observed trajectory. This process prevents estimates from being overly influenced by a small number of observations while still allowing meaningful individual differences to emerge. The resulting probabilities, therefore, reflect a balance between individual experience and population‐level evidence, producing stable estimates of future well‐being transitions while preserving heterogeneity across individuals. Figures 1 and 2 summarize the full estimation procedure, from data construction through to model estimation.3, 4, 5, 6, 7, 8, 9, 10

The third step in our estimation involves the estimation of psychological decision‐making parameters, that is, the parameters of CPT, which model how individuals psychologically evaluate gains, losses, and uncertainty. The CPT suggests that people do not treat gains and losses equally. Losses often have a stronger emotional impact than gains of the same size. Likewise, people may distort probabilities, giving too much weight to unlikely events and too little weight to more likely ones. Because individuals differ in these tendencies, we estimate a separate set of psychological parameters for each person. These parameters capture (i) *loss aversion*: how strongly someone reacts to losses relative to gains, (ii) *sensitivity to gains and losses*: how sharply people respond to changes in well‐being, and (iii) *probability weighting*: how people perceive and interpret uncertain future outcomes. To estimate these characteristics, we use a hierarchical Bayesian model. In simple terms, this method allows us to learn about each individual while also drawing on information from the broader sample when individual data are limited. This helps produce more reliable estimates than treating every respondent entirely independently.

Technically speaking, the subjective probability $p_{it}$ is the posterior probability that individual i will move to a strictly higher well‐being state next period, given current state $s_{it}$. Recalling the equation

(11) $$p_{it}\;=\;\sum_{s^{\prime }>s_{it}}P_{i}\left[s_{it},\;s^{\prime }\right].$$

We note that the values of $p_{it}$ are bounded to [0.02,0.98] for numerical stability. In the estimation sample, the mean subjective probability is 0.294. The within‐sample correlation between $p_{it}$ and the contemporaneous well‐being change $x_{it}$ is −0.476, suggesting that individuals who experienced declining well‐being assign substantially lower probabilities to future improvement. The near‐zero correlation between $p_{it}$ and SI (r=+0.046) suggests that raw transition probability alone is not strongly associated with ideation; the full CPT weighting structure is required.

A central contribution of this study is the estimation of *individual‐level* CPT parameters rather than population averages. We specify a fully hierarchical Bayesian model in which each individual's parameter vector $\theta_{i}$ is drawn from a population distribution with unknown hyperparameters, estimated jointly from the data. This partial‐pooling (shrinkage) structure recovers individual parameters where data are informative while regularizing toward the population in sparse‐data cases. To avoid the funnel geometry that impedes NUTS sampling in hierarchical models (Betancourt 2017), each CPT parameter is modeled through a noncentered reparameterization:

(12) $$\theta_{i}\;=\;\mu_{\theta }+\sigma_{\theta }⊙z_{i},\;z_{i}∼N\left(0,I\right),$$

where $\mu_{\theta }$ and $\sigma_{\theta }$ are population mean and standard deviation hyperparameters. Bounded parameters are recovered via inverse‐logit transformations. Population hyperpriors are centered on TK1992's empirical benchmarks λ≈2.25, α=β≈0.88, and γ=δ≈0.69 reflecting four decades of accumulated CPT evidence while remaining weakly informative enough to accommodate departures in our nonstandard domain.

**Linking Prospect Valuation to Suicide**: Once we have estimated these psychological parameters, we calculate a CPT‐based value score for each person and year. This score summarizes how an individual evaluates their recent well‐being changes, together with their expectations about the future. We then examine whether lower CPT values—reflecting perceived losses combined with pessimistic expectations—are associated with a greater likelihood of reporting SI. Technically, individual‐level CPT values $V_{it}$ enter a logistic regression linking behavioral valuation to SI:

(13) $$\mathrm{Pr}\left({\text{SI}}_{it}=1\right)\;=\;{\mathrm{logit}}^{-1}\;\left(\kappa +\beta_{\mathrm{cpt}}\cdot V_{it}\right),$$

where $\beta_{\mathrm{cpt}}$ is the central parameter of interest, expected to be negative: lower CPT value which reflecting perceived loss amplified by loss aversion and pessimistic probability weighting should increase SI probability. The prior on $\beta_{\mathrm{cpt}}$ is N(0,2), placing no directional presumption on the result. Demographic heterogeneity in the CPT–SI relationship is examined through subgroup average marginal effects (AMEs) stratified by gender and age group, reported in Section 5.5. The analysis, therefore, tests a central prediction of the theory: SI should be more strongly related to how individuals perceive and evaluate worsening circumstances than to objective circumstances alone. Our empirical strategy exploits within‐individual variation in well‐being over time, assuming that individuals serve as their own reference point. The intention to end life for an individual starts with the comparison of his own state of life lived thus far, for example, the low $V_{i,t-1}$ predicts suicide ideation, $S_{it}$. The past well‐being value of his life may as well capture not only his own but also a comparison of well‐being with others. While our strategy does not identify the causal effects strictly, the longitudinal structure of the data allows us to examine how changes in well‐being trajectories are associated with subsequent SI.

## 5 Empirical Results

### 5.1 Descriptive Statistics

Before turning to structural estimates, the raw data reveal a relationship of striking clarity. Individuals reporting SI had a mean Cantril Ladder score of 4.08, against 6.34 for those without a gap of 2.26 points, or approximately 1.4 standard deviations (Figure 5). Critically, the well‐being *trajectory* is equally telling: the mean annual change in ladder score among SI =1 observations is −0.716, against +0.038 for SI =0 observations. Those who experienced ideation were not merely at lower rungs; they were descending.

The gain–loss domain analysis formalizes this asymmetry (Figure 10, Panel A). Among person‐year observations in the gain domain ($x_{it}>0$), the SI rate is 1.41%. Among observations in the loss domain ($x_{it}<0$), the SI rate is 3.02%: **2.14 times** the gain domain rate (relative risk =1.35, $\chi^{2}$ p<0.001). This domain asymmetry is the empirical signature of CPT's loss aversion channel the psychological impact of losing ground is disproportionate to the benefit of equivalent gains.

**FIGURE 7:**
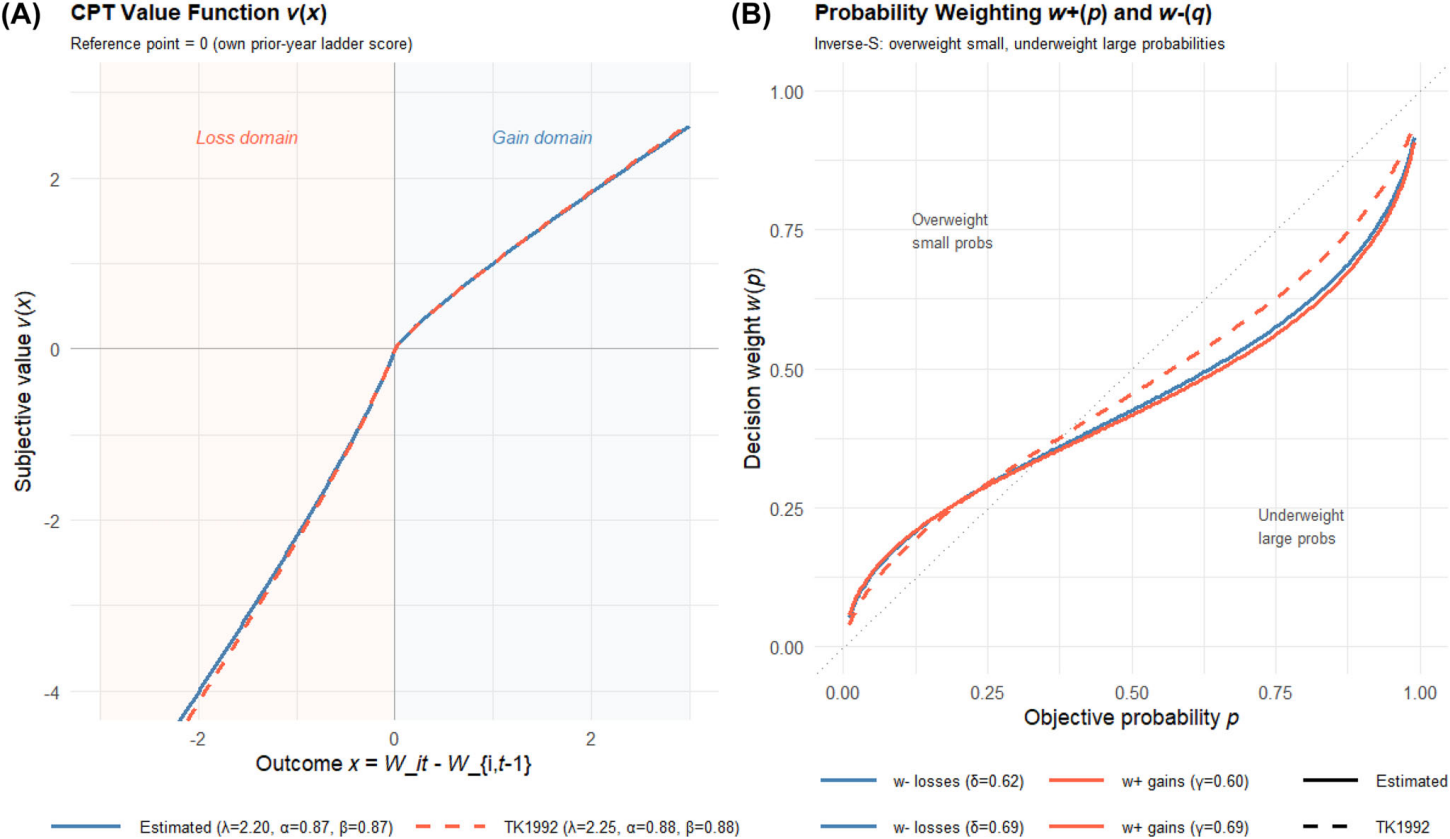
Estimated CPT value function and probability weighting curves vs. Tversky and Kahneman (1992) benchmarks. *Note*: Panel A shows the value function v(x) for gains and losses; Panel B shows the probability weighting functions $w^{+}\left(p;\hat{\gamma }\right)$ and $w^{-}\left(q;\hat{\delta }\right)$ alongside TK1992. The estimated curves replicate TK1992 with high fidelity in the value domain; probability weighting shows somewhat greater distortion (lower $\hat{\gamma }$ and $\hat{\delta }$) relative to TK1992's original laboratory estimates.

**FIGURE 8:**
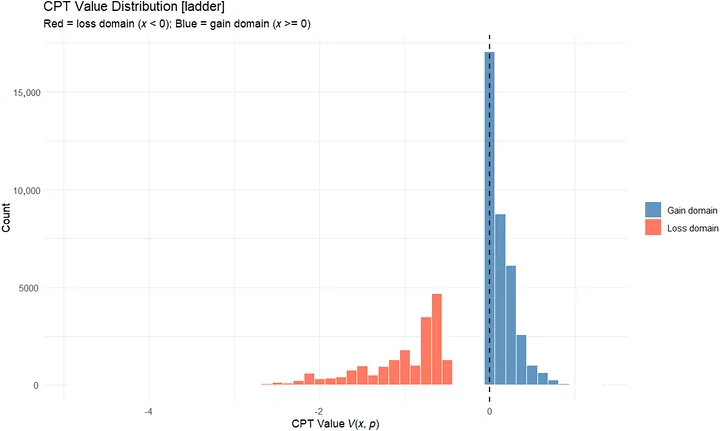
CPT value distribution (Ladder specification). *Note*: Gain domain ($x_{it}\geq 0$); loss domain ($x_{it}<0$). The dashed vertical line marks the reference point V=0. Gain domain observations cluster tightly near zero; loss domain observations exhibit a long left tail, reflecting loss aversion amplification of downward well‐being changes.

**FIGURE 9:**
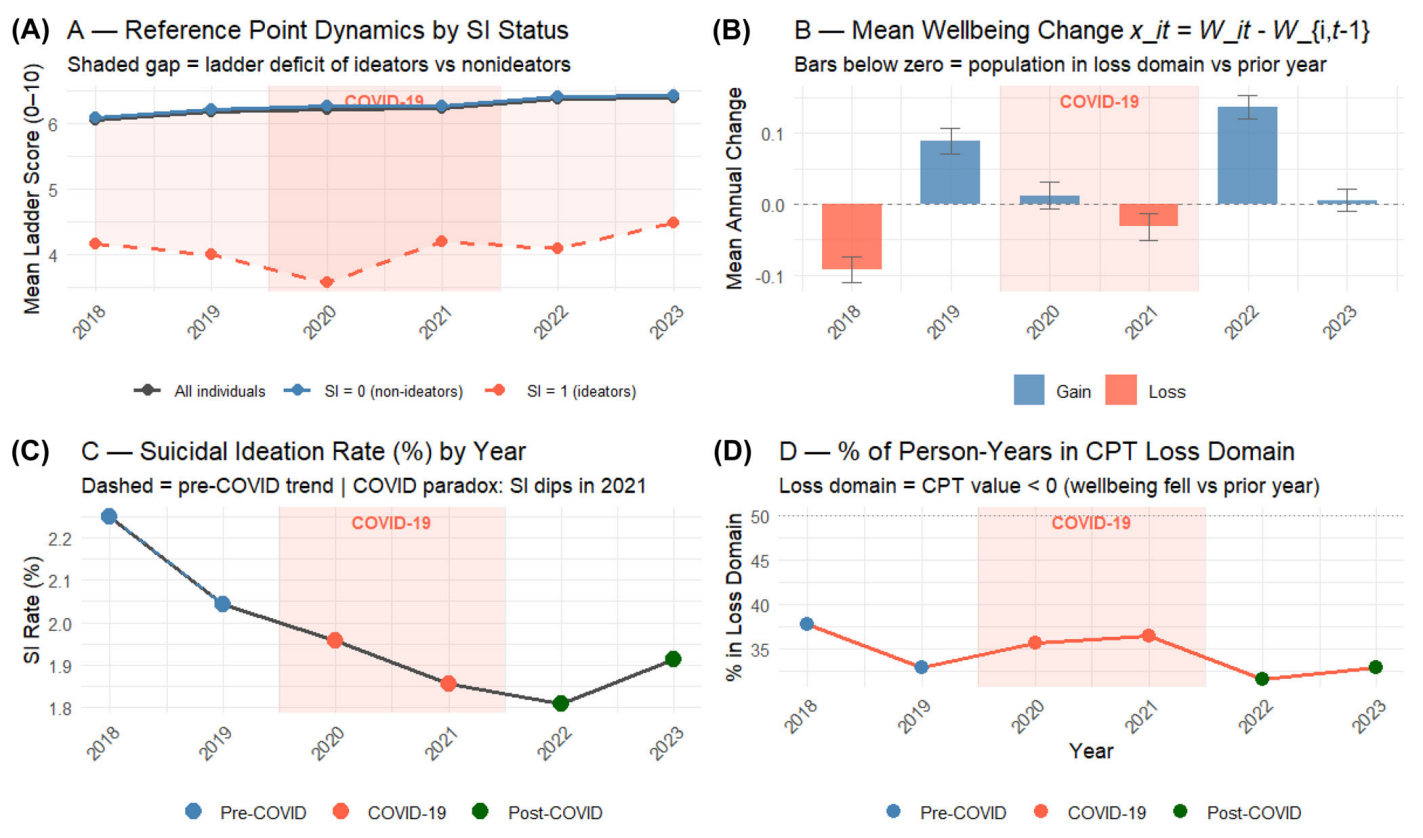
Temporal dynamics of well‐being, well‐being change, SI rate, and loss domain proportion (2018–2023). *Note*: Panel A: secular upward trend in mean Cantril Ladder with COVID‐19 disruption shaded. Panel B: mean annual well‐being change $x_{it}$; bars below zero indicate population‐level loss domain years. Panel C: SI rate by year; note the COVID paradox SI declines during 2020–2021 despite population‐level well‐being losses. Panel D: proportion of person‐years in the CPT loss domain; modest COVID‐period rise before returning to baseline.

**FIGURE 10:**
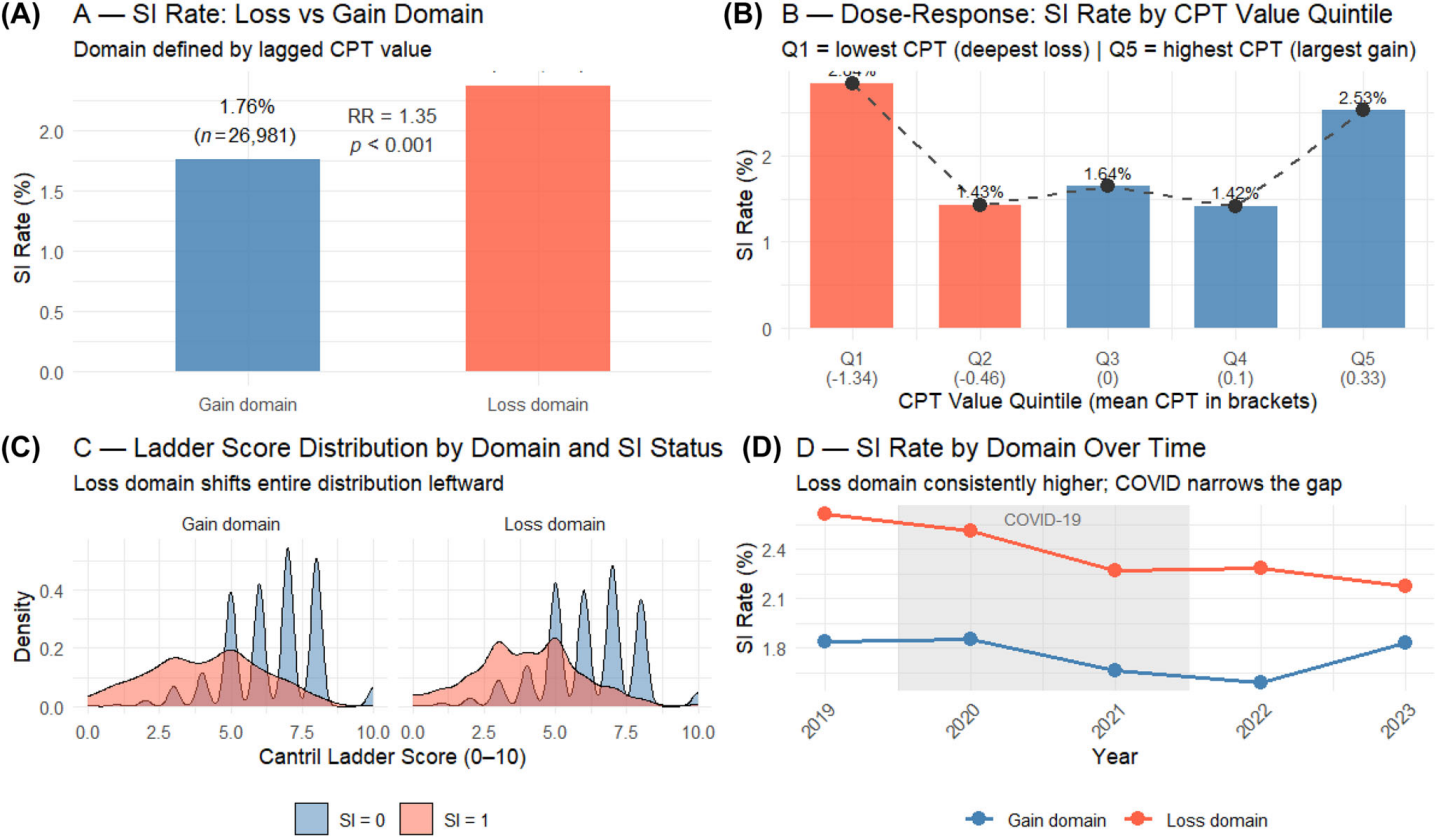
CPT domain analysis. *Note*: Panel A: SI rates by gain versus loss domain (lagged CPT value), with relative risk. Panel B: Dose–response SI rate by CPT value quintile; note the U‐shape with elevated risk at Q1 (severe loss) and Q5 (large gain). Panel C: Cantril Ladder distributions by domain and SI status; the loss domain shifts the SI =1 distribution leftward. Panel D: SI rate by domain over time; loss domain consistently higher, with COVID‐period narrowing.

A quintile analysis of CPT value reveals a further complication: the relationship is not monotonic (Figure 10, Panel B). Q1 (lowest CPT, deepest perceived loss) carries an SI rate of 2.84%; this declines sharply through Q2–Q4 (≈1.4%–1.6%) before rising again to 2.53% in Q5 (highest CPT, largest perceived gains). This U‐shaped pattern suggests two distinct risk pathways. The *primary* pathway runs through severe perceived loss: individuals in Q1 face downward well‐being trajectories amplified by loss aversion. The *secondary* pathway, concentrated in Q5, may reflect an aspiration‐reality mechanism: individuals who perceive large gains may face heightened sensitivity to future reversals, consistent with the overweighting of small probabilities in the gain domain generating volatile expectations attached to sharp upward trajectories. Both mechanisms are accommodated within the CPT framework.

The temporal evolution of these patterns is captured in Figure 9. While the Cantril Ladder exhibits a secular upward trend (6.18 in 2017 to 6.48 in 2023), the suicide ideation rate follows a declining trajectory, reaching a trough in 2022 before a modest post‐COVID rebound. The COVID‐19 period (2020–2021) produced negative mean annual well‐being changes the population moved into the loss domain yet the SI rate *declined* during these years. This *COVID paradox* (Figure 9, Panel C) is consistent with the social solidarity mechanism documented in the well‐being literature (Frijters et al. 2020): when losses are collectively experienced rather than individually idiosyncratic, the reference point shifts downward for the whole population, attenuating relative loss perception. The proportion of person‐years in the CPT loss domain rose modestly during COVID (from 34% to 37%) before returning to baseline.

### 5.2 Model Diagnostics and CPT Parameter Estimation

Prior to presenting the CPT parameter estimates and their implications, we present first, the results of model diagnostics. We observe that the convergence of the hierarchical model^16^ is robust across all scalar parameters. The maximum $\hat{R}$ is 1.0025 (well below the 1.01 threshold), the minimum bulk effective sample size is 2071 (well above 400), and there are zero divergent transitions and zero treedepth violations. The NUTS sampler explored the posterior geometry freely, confirming that the noncentered successfully resolved the funnel geometry that plagues centered hierarchical models with sparse individual data. Posterior predictive checks (Figure 3) confirm adequate model calibration: the observed SI rate of 1.97% falls within the 95% posterior predictive interval, with the mean simulated rate closely tracking the observed value.

We have estimated the CPT model using a hierarchical Bayesian framework, allowing individual‐level heterogeneity in behavioral parameters (the distribution of CPT parameters are presented in Figure 11). The estimated value function closely replicates the canonical parameterization reported in laboratory studies of prospect theory. In particular, the estimated value function exhibits the characteristic features of concavity in gains, convexity in losses, and a steeper slope in the loss domain, reflecting loss aversion (Figure A.1). Likewise, the estimated probability weighting functions display the familiar inverse‐*S* shape, indicating overweighting of small probabilities and underweighting of large probabilities. Relative to laboratory benchmarks, however, the estimated weighting functions show somewhat stronger distortion, suggesting that individuals facing real‐world socioeconomic uncertainty may exhibit greater behavioral deviations from expected utility (Figure 8).

**FIGURE 11:**
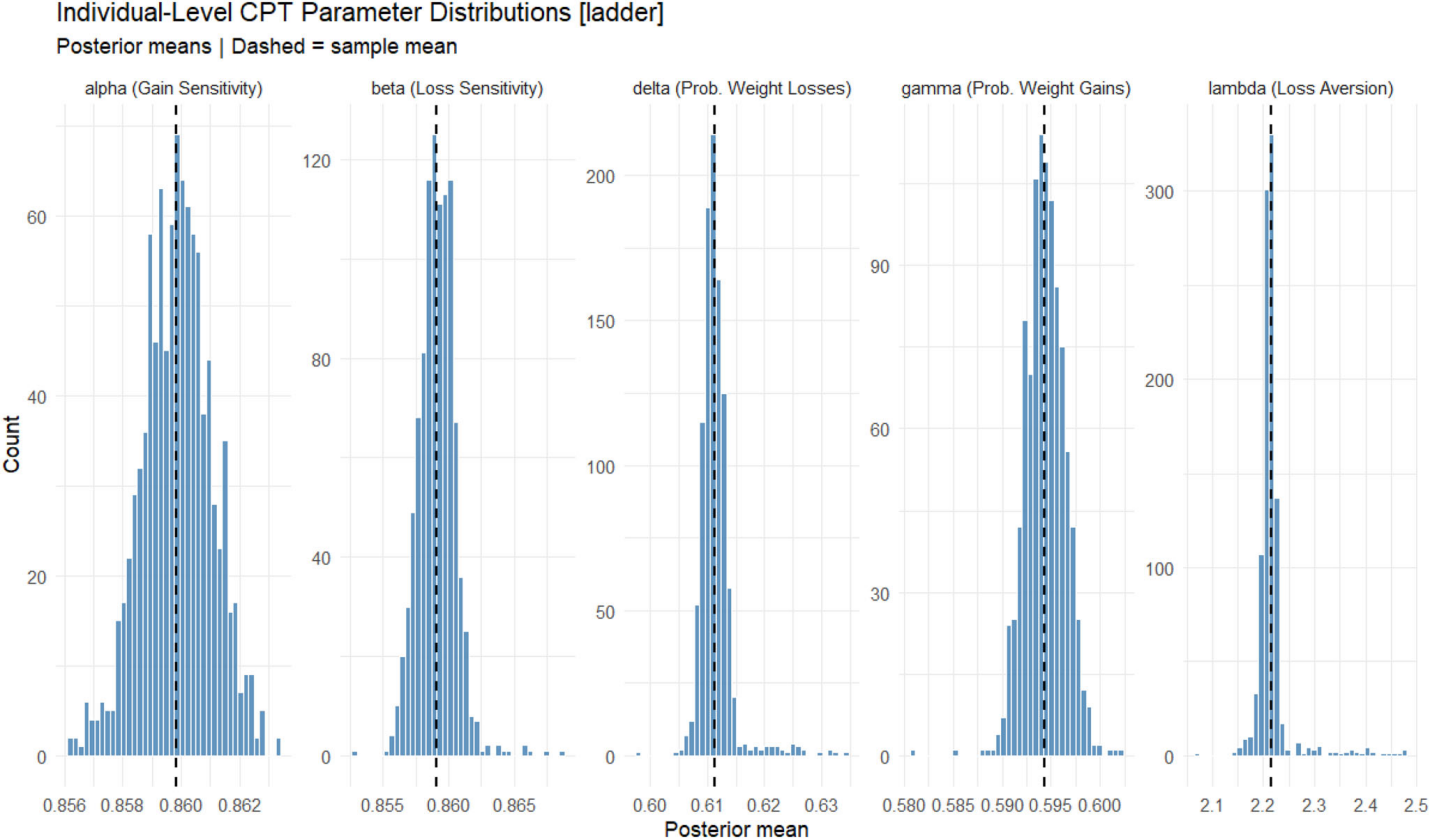
Individual‐level CPT parameter distributions (posterior means, N=1000). *Note*: Each panel shows the distribution of individual posterior means for one CPT parameter; dashed lines indicate the sample mean. Tight clustering around population means reflects hierarchical shrinkage under sparse individual data (mean 3.8 observations per person). Loss aversion λ exhibits the widest cross‐individual spread, consistent with its role as the primary driver of suicide ideation heterogeneity.

**FIGURE 12:**
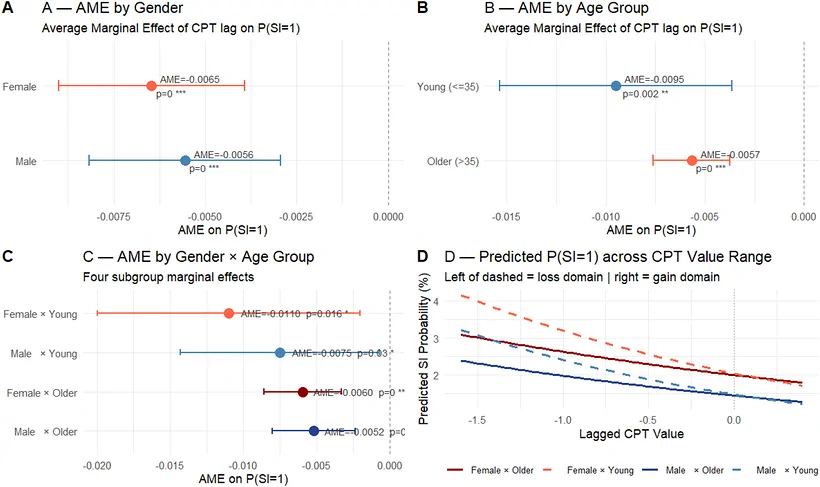
Average marginal effects of lagged CPT value on Pr(SI=1). *Note*: Panel A: AME by gender; Panel B: AME by age group; Panel C: AME by gender × age interaction (Female × Young is largest in absolute magnitude); Panel D: predicted SI probability across the lagged CPT value range by demographic subgroup, with loss domain (left of dashed) and gain domain (right) distinguished. All AMEs are negative; all 95% CIs exclude zero.

Table 2 reports posterior means and 90% credible intervals for the population CPT hyperparameters alongside Tversky and Kahneman (1992) benchmarks. The South Korean panel yields a loss aversion coefficient ($\hat{\lambda }=2.197$) nearly identical to Tversky and Kahneman (1992) estimate of 2.25, derived from laboratory experiments with monetary gambles. Value function curvature ($\hat{\alpha }=\hat{\beta }=0.872$) likewise closely tracks Tversky and Kahneman (1992)'s 0.88. This cross‐cultural, cross‐domain replication, from financial gambles in the laboratory to life satisfaction trajectories in a nationally representative panel is a substantive result in its own right, suggesting that the psychological architecture of prospect theory is a robust feature of human cognition rather than a laboratory or domain‐specific artifact.

The probability weighting parameters ($\hat{\gamma }=0.601$, $\hat{\delta }=0.618$) are modestly below Tversky and Kahneman (1992)'s estimates of 0.69, indicating somewhat stronger probability distortion in our sample. Whether this reflects a cultural difference in probability perception, a feature of the life satisfaction domain, or an interaction with our Bayesian Markov probability construction is a question that the robustness specifications address. Figure 7 illustrates how closely the estimated curves track Tversky and Kahneman (1992) in both the value function and the probability weighting plane.

### 5.3 Prediction of Suicide Ideation by Prospect Valuation

We next examine whether prospect‐theoretic valuations predict SI in the logistic outcome model. The central coefficient of interest is ${\hat{\beta }}_{\mathrm{cpt}}=-1.010$ (90% CrI: [−1.63,−0.597]). The entire credible interval lies strictly below zero. This establishes that lower CPT value a perception of well‐being loss, amplified by the nonlinear loss aversion and probability weighting mechanisms of CPT, systematically and robustly increases the probability of SI. The direction was not imposed by the prior, which was centered at zero.

We estimate the parameters using generalized estimating equation (GEE) that uses a generalized linear model with a possible unmeasured correlation between observations from different time points. The GEE panel estimate provides population‐average quantification with causal temporal ordering (Table 3). A one‐unit increase in lagged CPT value reduces SI odds by 18.5% (OR =0.815, 95% CI: [0.727,0.915], p=0.0005). The use of lagged CPT value well‐being trajectory at t−1 predicting SI at t provides a natural temporal ordering that strengthens the causal interpretation without requiring an instrumental variable. The COVID‐19 year indicator is small and statistically null (estimate: −0.027, p=0.805), confirming that the pandemic generated no independent SI shock over and above what is captured by the CPT value channel: aggregate adversity shifts the reference point for everyone, attenuating individual loss perception precisely the Adaptation Level mechanism (Helson 1964).

To summarize, we find that consistent with the theoretical predictions, the estimated coefficient on the CPT valuation index is negative and statistically significant. Lower prospect values—reflecting perceived losses amplified by loss aversion and pessimistic probability weighting—are associated with a higher probability of SI. Importantly, subjective transition probabilities alone are not strongly associated with SI. The correlation between the probability of future well‐being improvement and SI is close to zero (r=0.046). This finding indicates that expectations about the future influence SI primarily through their interaction with reference‐dependent valuation rather than as stand‐alone predictors.

### 5.4 Which CPT Parameters Drive Suicide Risk?

To identify the behavioral mechanisms driving the results, we examine the contribution of individual CPT parameters to suicide risk. The hierarchical model produces individual‐level posterior means for each of the five CPT parameters. Table 4 reports Pearson correlations between these individual‐level estimates and individual mean SI rates. The results are unambiguous: it is the *loss‐side parameters that govern suicide ideation risk*. Loss aversion (λ) carries a correlation with individual SI rates of r=+0.629 (p<0.001). Probability weighting for losses (δ) carries r=+0.554 (p<0.001). Loss sensitivity (β) contributes a significant but modest r=+0.146 (p<0.001). In contrast, gain sensitivity (α) is essentially orthogonal to SI risk (r=+0.030, p=0.337), and probability weighting for gains (γ) is weakly negatively correlated (r=−0.072, p=0.022). The gain domain parameters contribute nothing to individual variation in SI risk. It is not how individuals process gains but how severely they process losses that determines their position on the SI distribution.

The clinical salience of loss aversion is sharpened by the quartile analysis (Figure 4). Dividing individuals into quartiles by estimated λ, the Q4 (high loss aversion) group exhibits a mean SI rate of 5.80%, 12.4 times higher than Q1 (0.47%). Risk does not rise gradually: Q1 through Q3 cluster below 0.70%, with exposure concentrated almost entirely in Q4. High loss aversion is not a quantitative increment in SI risk; it marks a qualitative threshold beyond which reference‐dependent well‐being losses translate into acute psychological distress. This finding has direct implications for population screening: individuals with psychologically high loss aversion constitute a disproportionate share of SI risk and may be identifiable through behavioral or survey‐based elicitation before ideation crystallises.

To conclude, our analysis reveals that loss aversion plays the dominant role. Individuals characterized by stronger loss aversion exhibit substantially higher sensitivity to negative well‐being shocks, leading to larger declines in prospect when entering the loss domain. By contrast, variation in gain curvature contributes relatively little to the prediction of SI. This asymmetry reinforces the interpretation that psychological responses to losses, rather than gains, constitute the primary behavioral channel linking socioeconomic experiences to SI.

### 5.5 Demographic Heterogeneity: AMEs

Finally, we examine whether the relationship between prospect valuation and SI varies across demographic groups. The AMEs of the lagged CPT value on $\mathrm{Pr}({\mathrm{SI}}_{it}=1)$ are computed from a GEE logit model stratified by gender and age group (Table 5; Figure 12). The full‐sample AME is −0.0061 (SE =0.0009, p<0.001): a one‐unit increase in lagged CPT value reduces SI probability by 0.61 percentage points, a 31% relative reduction on the base rate of 1.97%. The AMEs indicate that the behavioral effects of well‐being losses are present across demographic categories but differ in magnitude.

Two patterns dominate. First, the *age gradient* is the primary axis of heterogeneity: young people (age ≤35) face an AME of −0.0095, nearly 1.7 times larger in absolute magnitude than the older group's −0.0057. A one‐unit increase in lagged CPT value reduces suicide ideation probability by 0.95 percentage points among the young, a 48% reduction on their base rate. This heightened sensitivity is theoretically coherent: younger individuals have shorter histories of adverse experience, less developed coping repertoires, and steeper reference‐dependent reactions to downward well‐being transitions, consistent with the developmental psychology literature on adolescent and young adult vulnerability to perceived failure (Nolen‐Hoeksema 2012). The results suggest greater vulnerability to loss‐domain dynamics. These findings are consistent with broader evidence linking socioeconomic insecurity and relative deprivation to mental health outcomes.

Second, the gender–age interaction identifies *young women as the most vulnerable demographic subgroup*. The AME for Female × Young is −0.0110 the largest in absolute magnitude across all subgroups and nearly double the full‐sample effect. The predicted SI probability for this group extends above 4% at the lowest CPT values (Figure 12, Panel D), well above the sample mean. This finding is consistent with the literature on gender differences in internalising versus externalizing responses to stress (Nolen‐Hoeksema 2012) and with South Korea‐specific evidence on the distinctive psychological burden borne by young women in highly competitive social environments. Critically, the CPT‐Suicide ideation gradient is uniformly negative across all subgroups, with all AMEs statistically distinguishable from zero, confirming that the mechanism operates population‐wide with differential intensity rather than being confined to a demographic niche.

### 5.6 Reverse Causality

Identification concerns may arise with regard to the causal interpretations because SI may itself depress subsequent subjective well‐being through social withdrawal, reduced labor force participation, or the psychological sequelae of distress so that the lagged CPT value partly reflects prior ideation rather than independently predicting it. We attempt to address this through three tests^17^ reported in Tables 6 and 7.

The first two tests exploit the COVID‐19 period as a source of exogenous variation in subjective well‐being. Pandemic‐driven deteriorations in life satisfaction during 2020–2021 reflected aggregate societal disruption unrelated to individual psychological histories, substantially attenuating the scope for prior SI to have generated the observed well‐being declines. Estimating the baseline GEE separately for COVID and non‐COVID waves yields odds ratios of 0.765 and 0.806, respectively, against the full sample baseline of 0.815, a stability ratio of 0.949, indicating near‐identical effect magnitudes across periods with and without the reverse causation channel (Panel A, Table 6). Formalizing this comparison via a CPT × COVID interaction term confirms the visual impression: the interaction odds ratio is 1.022 (95% CI: [0.804, 1.300], p=0.858), statistically indistinguishable from zero (Table 7). Reverse causation would predict a significant positive interaction with the CPT slope should flatten precisely when individual ideation cannot plausibly drive aggregate well‐being shocks, but no such attenuation is observed. The marginal significance of the COVID‐period CPT effect (p=0.060) reflects the smaller subsample size rather than any genuine weakening of the association.

The third test restricts the estimation sample to person‐years with ${\text{SI}}_{i,t-1}=0$, directly eliminating the persistence channel through which prior ideation could have depressed subsequent well‐being. Among 28,042 person‐years where individuals were not ideating in the prior wave, the CPT effect survives at OR =0.795 (p=0.004), essentially unchanged from the full‐sample baseline of 0.815 (Panel A, Table 6). Prior ideation cannot have generated the well‐being deterioration captured by $V_{i,t-1}$ in this window because there was none. In conclusion, we do not rule out reverse causation no observational design can but the three tests greatly limit its plausible role and support viewing the CPT–SI relationship as primarily prospective.

## 6 Discussions and Conclusions

This paper develops a behavioral framework for understanding SI by integrating subjective well‐being dynamics, loss aversion, and expectations about the future within a prospect‐theoretic perspective. Moving beyond conventional approaches that focus on static levels of well‐being, we show that individuals experiencing SI are not only worse off on average but are also more likely to be undergoing a decline in well‐being relative to their own past circumstances. This suggests that changes in well‐being may provide an important early warning signal for psychological vulnerability.

Our findings highlight a behavioral pathway linking adverse life experiences to SI. Negative changes in well‐being move individuals into a psychological “loss domain,” where losses are weighted more heavily than equivalent gains. Loss aversion then amplifies the impact of these experiences, while pessimistic expectations about future improvement further deepen negative evaluations of life prospects. SI appears most likely when declining well‐being, heightened sensitivity to losses, and diminished expectations about the future coincide. Our results show that observations in the loss domain exhibit more than twice the rate of SI observed in the gain domain, providing strong support for the behavioral prediction that losses loom larger than gains. The estimated prospect value functions confirm this asymmetry. Negative changes in well‐being generate substantially larger changes in prospect valuations than equivalent positive changes. This behavioral amplification mechanism may help explain why relatively moderate declines in socioeconomic circumstances, such as job loss, relationship breakdown, or financial stress, can produce disproportionately large psychological consequences for some individuals. In this sense, loss aversion may function as a behavioral multiplier, magnifying the mental health effects of adverse life events.

The results carry important implications for both research and policy. They suggest that suicide prevention efforts may benefit from monitoring trajectories of well‐being rather than relying solely on static indicators of mental health or socioeconomic status. Interventions that help individuals manage losses, adapt to adverse life events, or maintain hope for future improvement may be particularly effective in reducing risk. More broadly, the findings demonstrate how behavioral economic concepts can help explain why similar life shocks affect individuals differently and why seemingly moderate setbacks can sometimes produce severe psychological consequences.

Although the empirical analysis focuses on South Korea, the underlying behavioral mechanisms are likely to extend beyond any single national context. The tendency to evaluate life relative to personal reference points, to react more strongly to losses than gains, and to form judgments under uncertainty appears to be a general feature of human decision‐making. Future research could extend this framework by incorporating directly measured expectations about future well‐being, allowing a more precise understanding of how beliefs, adaptation, and uncertainty shape mental health outcomes over the life course.

## Figures and Tables

**FIGURE A.1:**
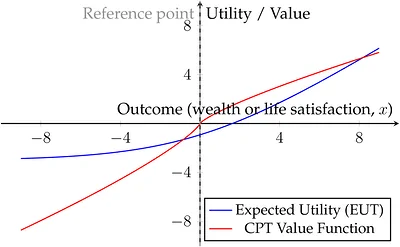
Comparison of Expected Utility and Cumulative Prospect Theory value functions. EUT assumes a smooth, concave utility over total outcomes. CPT introduces reference dependence, concavity for gains, convexity for losses, and steeper slope for losses (loss aversion).

## Endnotes

1 Although the theoretical treatment of suicide varies between ideation/planning and attempts, most empirical studies focus on suicide deaths. Ideation spans passive thoughts (e.g., wishing to be dead) to active planning (e.g., seeking methods or writing notes). When individuals perceive their current adverse circumstances as irreversible, these behaviors may progress to a fatal outcome. However, combining ideation and planning with suicide deaths in empirical analysis risks conflating distinct stages of the process and obscuring the specific mechanisms and triggers that lead from thoughts to action.

2 A recent World Health Organization (WHO) report shows that more than 727,000 people *commit* suicide every year around the world and millions more *attempt* to take their own lives. This results in an irreversible loss of the present value of the deceased's future earnings, adjusted for factors such as age, life expectancy, and employment rates. https://www.who.int/news‐room/fact‐sheets/detail/suicide.

3 Our work is related to Bjorklund (2023), who establishes a robust relationship between economic precarity and suicidal deaths, citing, for instance, that the white working class has experienced a surge in sociocultural isolation and disengagement with societal institutions.

4 This is in contrast to the *HS*'s EUT‐based predictions that suicide is a rational choice of individuals and that it grows with age and decreases with rise in income. Lepori et al. (2024), among others, provide counterfactual evidence.

5 Smith (2019) is an excellent commentary on the most glaring pitfalls of utility‐based models of *HS* (assumption of rationality in suicide choice), which have been somewhat tacitly followed or assumed in several empirical work including over 1500 research published in this journal alone).

6 While a richer implementation incorporating exponentially discounted accumulation of past shocks (Barberis 2013) would more fully capture deep memory effects, it would require substantially longer panel histories than the six‐wave Korean Welfare Panel Study (KoWePS) window permits; we therefore treat our first‐difference specification as a theoretically grounded lower bound on the memory effect.

7 One can use the same logic to predict suicidal planning and attempt as well.

8 The authors showed that (ex post) attempters have higher incomes than those who seriously considered suicide but did not attempt.

9 Many survivors have noted superior well‐being following the incident. We will present some evidence in the empirical section.

10 In our empirical setting, gains and losses are comparable because both are measured as one‐unit movements on the same 0–10 Cantril Ladder scale. Thus, loss aversion does not compare qualitatively different objects such as income, health, and relationships directly. It compares equal‐sized upward and downward movements in the same subjective well‐being metric. For instance, a one unit decline from 6 to 5 and a one unit improvement from 5 to 6 are measured in the same unit, but the CPT value function allows the decline to carry greater psychological weight than the improvement.

11 Figure B.1 in Appendix B presents a graphical comparison of the EUT and CPT value functions.

12 We thank Pak and Choung (2020) for offering help and Korea Institute for Health and Social Affairs for providing us the detailed data.

13 A challenge we faced regarding the labels of the variables. The source data set is provided exclusively in Korean, limiting accessibility for the international research community and impeding reproducible cross‐linguistic scholarship. We addressed this through a systematic translation workflow using the Google Translation API, implemented via a fully scripted R pipeline. Rather than relying on ad hoc manual translation, which introduces semantic drift and inconsistency, the automated approach ensured uniform treatment of all 2798 variable labels, with contextual meaning validated by cross‐referencing semantically related variables within conceptual clusters.

14 The two most common discretization methods are Binary/Threshold Classification (standard in clinical or youth‐health studies) and World Happiness Report (WHR) Tiers.

15 Figures 1 and 2 describe our methodological flowchart—from sample and data construction to model building and estimation.

16 Stan Hierarchical model provides a general‐purpose sampler using Hamiltonian Monte Carlo, and it automates the required computation allowing us to conduct Bayesian inference by focusing solely on model building.

17 Note that the causal effects presented here need to be interpreted with caution, given the limitations of data and hence the approach we have adopted to mitigate endogeneity concerns.

## Appendix A

*HS* model extends the classical von Neumann–Morgenstern paradigm to an individual's life horizon. The expected lifetime utility at age a is defined as:

(A.1)
$$Z\left(a,Y_{P}\right)=\int_{a}^{\omega_{0}}e^{-r(m-a)}U\left[C\left(m,Y_{P}\right)-K\left(m\right)\right]P\left(m\right)\;dm,$$

where $Y_{P}$ denotes permanent income, K(m) the cost of subsistence, r the discount rate, and P(m) the probability of survival to age m. In this framework, an individual commits suicide when expected lifetime utility plus a subjective “taste for living” term $b_{i}$ equals zero:

(A.2)
$$Z\left(a,Y_{P}\right)+b_{i}=0.$$

Here, $Y_{P}$ is the permanent income. In the *HS* framework, suicide rates, *S*, respond monotonically to variations in macroeconomic variables:

$$S\left(a,Y_{P}\right)=f\left(a,Y_{P},U_{n}\right),$$

where unemployment and lower income reduces expected lifetime utility. Differentiating *S* with respect to age (*a*) and permanent income ($Y_{P}$) yield:

$$\frac{\partial S}{\partial a}>0,\;\frac{\partial S}{\partial Y_{P}}<0,\;\frac{\partial^{2}S}{\partial Y_{P}^{2}}>0.$$

## Appendix B

**TABLE B.1 risa70334-tbl-0008:** A synthetic map of the literature.

Utility‐based lens (EUT / HS‐lineage)	Middle bridge: SES → learning/memory → stage transitions	Prospect‐theory / CPT + computational lens
***Core framing***		
Suicide/attempt as a choice driven by discounted lifetime utility; “distaste for life” and expected future utility (Hamermesh and Soss 1974).	Socioeconomic trajectories shift reference points and perceived option sets; repeated shocks accumulate into memory, altering updating and the perceived viability of “search” for alternatives (learning failure). Stage outcomes must be separated (ideation, planning, and attempt) (Klonsky et al. 2016; O'Connor and Kirtley 2018; Joiner et al. 2009).	Reference dependence, loss aversion, diminishing sensitivity, and nonlinear probability weighting govern valuation under uncertainty (CPT tradition; use your PT/CPT key: tversky).
***Attempts and transitions***		
Attempts are not necessarily terminal: modeling survival and postattempt utility implies attempts may occur when conditional‐on‐survival prospects improve (Marcotte and Hansen 2024). Youth attempts may be strategic signaling; contagion and family structure matter (Cutler et al. 2001).	SES stressors can produce defeat/entrapment (motivational) and interact with volitional constraints (capability/means/planning) to govern transitions (O'Connor and Kirtley 2018; Joiner et al. 2009). Learning failures can appear as persistent pessimistic updating or reduced adaptation to feedback, increasing the salience of losses and narrowing perceived alternatives.	Attempt‐history groups show altered valuation under risk/loss: heightened aversion to risk and loss (Baek et al. 2017); higher loss aversion in attempters vs. nonattempters (Liu et al. 2022). Loss aversion may be stage‐specific, predicting attempts but not ideation (Hadlaczky et al. 2018).
***Social comparison and environment***		
Relative standing matters in economic hazards: suicide risk rises with others' income holding own income fixed (Daly et al. 2013). Method choice is dynamic; restricting a method may induce substitution across timing/methods (Whitman 2002).	Macro/SES environments pattern risk: inequalities and vulnerability can widen following macro stress (Lorant et al. 2005; Hiyoshi et al. 2018; Lepori et al. 2024). Relative deprivation predicts ideation and planning/attempt above absolute income (Pak and Choung 2020). Policy protection (minimum wage) reduces suicide among lower‐education groups, especially under high unemployment (Kaufman et al. 2020). Scarcity imposes cognitive load that can reduce effective control and degrade learning/decision quality (Mani et al. 2013).	CPT makes reference points explicit: peer/past‐self benchmarks map directly into reference‐dependent losses; distorted beliefs about uncertain futures are representable via probability weighting (and can vary with symptom severity) (Hagiwara et al. 2022).
***Learning, adaptation, and prediction***		
Learning is typically implicit or reduced‐form in EUT suicide models; prediction often relies on broad correlates. Critiques argue the HS backbone is empirically fragile and conceptually incomplete as a general theory (Smith 2019).	Prediction remains difficult, motivating mechanism‐linked constructs and explicit transition modeling (Ribeiro et al. 2015). A key missing link is a population‐scale integration: SES trajectories → (reference points + learning/memory) → stage‐specific outcomes.	Decision‐neuroscience emphasizes valuation/learning disruptions in suicidality (Dombrovski and Hallquist 2017; Dombrovski et al. 2013). Compact reward/aversion judgment tasks can predict distinct STB outcomes (notably planning) with stage‐specific feature importance …(Lalvani et al. 2024).
***Our contribution (this paper)***		
HS provides a tractable benchmark but is insufficient as a general backbone under heterogeneity and critique (Hamermesh and Soss 1974; Smith 2019).	We operationalize SES‐based reference points (peer/time benchmarks), construct CPT‐style indices from longitudinal SES trajectories, and embed learning with memory (adaptive/Bayes/RL). We estimate stage‐specific models (ideation, planning, attempt) consistent with ideation‐to‐action frameworks and test incremental predictive value out‐of‐sample (Klonsky et al. 2016; O'Connor and Kirtley 2018; Ribeiro et al. 2015).	We ground behavioral primitives in CPT/computational evidence (loss aversion, probability weighting, learning/adaptation) and link them to socioeconomic processes rather than treating them as purely clinical lab traits (Baek et al. 2017; Hadlaczky et al. 2018; Liu et al. 2022; Hagiwara et al. 2022).

We can easily compare the EUT and CPT value functions via Figure A.1 below:[Table risa70334-tbl-0008]
